# Identifying Nurses at Risk of Nursing Interruptions During Medication Administration Using Machine Learning: A Multicenter Cross‐Sectional Study

**DOI:** 10.1155/jonm/4433675

**Published:** 2026-04-20

**Authors:** Xiaoqian Dong, Siqing Ding, Sha Wang, Min Liu, Gang Gan, Huan Cao, Xingxing Wang, Nandan Chen, Bangdi Tan, Jianfei Xie, Peter H. F. Ng

**Affiliations:** ^1^ Nursing Department, Third Xiangya Hospital, Central South University, Changsha, 410000, Hunan, China, csu.edu.cn; ^2^ Xiangya School of Nursing, Central South University, Changsha, 410031, Hunan, China, csu.edu.cn; ^3^ Nursing Department, Changsha Jingkai Hospital, Changsha, 410031, Hunan, China; ^4^ Clinical Nursing Safety Management Research Center, Central South University, Changsha, 410031, Hunan, China, csu.edu.cn; ^5^ Nursing Department, Dongguan Humen Hospital, Dongguan, 523939, Guangdong, China; ^6^ Department of Computing and Department of Rehabilitation Science, The Hong Kong Polytechnic University, Hong Kong, China, polyu.edu.hk

**Keywords:** machine learning, medication safety, nurses, nursing interruption, risk prediction model

## Abstract

**Background and Aims:**

Nursing interruptions during medication administration (NIMA) critically contribute to medication administration errors. However, validated prediction tools for NIMA risk assessment are unavailable. This study aimed to develop and internally validate three machine learning−based prediction models (logistic regression [LR], decision tree [DT], and Naive Bayes [NB]) for identifying nurses’ individualized risk of NIMA.

**Methods:**

A total of 4758 Chinese nurses were recruited from 12 tertiary hospitals between November 2023 and January 2024. The outcome was defined as the occurrence of ≥ 1 NIMA during the latest work shift. We used univariate analysis and LR to identify predictors. Participants were randomly allocated to training (*n* = 3806; 80%) and internal validation (*n* = 952; 20%) sets. Three machine learning models were implemented in Python, with performance evaluated via 1000‐iteration stratified bootstrapping. Key performance metrics included AUC, accuracy, recall, specificity, precision, F1‐score, and G‐mean. Models comparisons used the DeLong test. The best model was further assessed with the Hosmer−Lemeshow test and calibration curves.

**Results:**

Overall, 52.1% of nurses experienced at least one NIMA event. Predictive features of NIMA included 18 factors, such as department type, marital status, shift type, general self‐efficacy level, and the needs of multiple people. In the test set, AUCs ranged from 0.679 to 0.748. The LR model performed best, achieving the highest AUC of 0.748 (95% CI: 0.717–0.779), an accuracy of 0.694 (95% CI: 0.664–0.724), and other performance metrics, including precision, recall, specificity, F1‐score, and G‐mean. The calibration of the LR model was supported by the Hosmer−Lemeshow test (*X* ^2^ = 7.062, *p* = 0.530).

**Conclusions:**

This study systematically evaluated multiple influencing factors of NIMA and developed three internally validated risk prediction models of NIMA. The LR‐based nomogram and the web‐based calculators showed the most consistent performance and may support risk stratification and targeted nursing management, pending external validation and feasibility assessment.

## 1. Introduction

Patient harm from unsafe care remains a major global public health challenge. The World Health Organization (WHO) reports that around 1 in every 10 patients is harmed in healthcare, and more than 3 million deaths occur annually due to unsafe care [1]; these figures reflect unsafe care overall rather than medication administration errors (MAEs) specifically. WHO further notes that medication‐related harm accounts for a substantial share of preventable patient harm and estimates that medication errors impose an annual global economic burden of approximately US $42 billion [2]. Within this broader context, MAEs, errors occurring at the administration stage, remain a common and preventable source of patient harm. Beyond mortality, MAEs prolong hospitalizations, inflict physical harm, and cause profound psychological, social, and financial consequences for patients [3]. Nurses, as frontline professionals responsible for medication administration, are implicated in 21%–59% of MAEs [4], with approximately 43% of these errors being attributed to nursing interruptions (NIs) [5]. NI refers to a sudden external behavior that interrupts or delays the ongoing nursing procedures and distracts nurses’ attention from the delivery of care for patients under a specified time, role, and environment [6, 7]. Previous studies have shown that 10%–66% of nurses are interrupted during medication administration [8]. Crucially, each NI during the medication administration (NIs during medication administration [NIMA]) raises the risk of medication error by 12.7%–13% [9]. Therefore, NIMA is a primary modifiable risk factor in managing MAEs.

However, NIMA frequently occurred among nurses in clinical settings, including emergency departments [9], pediatrics [10], and the ICU [5], among others. A prior study reported that the rate of NIMA among pediatric nurses reached 94.51% [10]. Therefore, early identification of nurses at risk of NIMA is essential for preventing medication‐related nursing adverse events. Unfortunately, there is a lack of large‐scale data to predict NIMA and to establish appropriate surveillance strategies based on risk stratification. This is due to the reliance on structural observation for NIMA measurement, which is labor‐intensive, and the current self‐designed observational tools capture only limited and common influencing factors, making it difficult to develop comprehensive predictive models [11–14]. Recently, although two self‐assessment tools are available for investigating NIs, including “Workplace Interruptions Measure Scale” [15] and “Nursing Work Interruption Scale” [16], neither focused on the specific situation of medication nursing. In addition, dimensions of the Nursing Work Interruption Scale were limited to human and environmental factors only, which made it difficult to comprehensively reflect the multidimensional causes of NIMA. Several external factors, such as patient or family demands, colleague inquiries, and environmental noise [12, 17], have been consistently linked to NIMA. In addition, nurse internal factors such as mental workload and time pressure have shown significant associations with increased NIMA risk [12, 13, 18]. We applied the theoretical framework of the systemic accident causation model based on safety information flow [19] and systematically identified NIMA risk factors across six dimensions: human, environment, resources, equipment, information, and management. Then, we developed a structured indicator system, providing a practical tool for the comprehensive identification and quantification of NIMA risk factors [20].

Among the various factors associated with NIMA, identifying those that confer the greatest risk is essential. Leveraging these key predictors, predictive models can help detect vulnerable nurses and guide more targeted intervention strategies. Prediction models have proven successful in managing patient safety issues, such as unplanned extubation and catheter‐related thrombosis [21, 22]. In particular, machine learning algorithms provide powerful advantages over traditional statistical approaches by processing high‐dimensional data and uncovering complex, nonlinear relationships between predictors [23] and are increasingly used in safety prediction research [24]. Furthermore, nomograms transform traditional predictive models into easy‐to‐use numerical probabilities, facilitating clinical assessment and decisions in nursing management [25]. Despite these advances, studies specifically focusing on the risk prediction of NIMA remain scarce, and few tools have been developed to support NIMA risk assessment. Therefore, this study aims to (1) examine and determine factors influencing NIMA; (2) develop and evaluate machine learning algorithm‐based risk predictive models for NIMA in nurses; and finally, (3) create a user‐friendly nomogram to estimate NIMA risk.

## 2. Methods

### 2.1. Study Design, Setting, and Participants

From November 2023 to January 2024, we conducted a cross‐sectional survey to investigate clinical nurses in 12 hospitals of central China using convenience sampling. Prior to data collection, we obtained permission from the directors of nursing departments at each participating hospital. With their assistance, eligible nurses were identified according to the inclusion and exclusion criteria within their respective departments. Inclusion criteria were as follows: (a) holding a registered nurse license, (b) full‐time employment with a minimum of one year of clinical experience, and (c) working in the current department for not less than 3 months. We excluded some nurses, such as those who came to visit and study; those who could not participate due to personal leave, maternity leave, sick leave, study away, and other reasons; and nurses whose work did not involve medication operation (disinfection supply center and other positions). Written informed consent was obtained from all participants, who were assured of their right to withdraw. All data were kept confidential. The Ethics Committee of Xiangya School of Nursing, Central South University (E2022184), approved the study.

### 2.2. Outcome Assessment

The outcome was defined as the occurrence of ≥ 1 NIMA event during the latest work shift. In view of the lack of standardized NIMA self‐assessment tools in existing studies, this study adopted a multiphase hybrid approach for event identification and data collection, with the specific process as follows: (1) NIMA cognitive standardization training: We conducted situational cognitive training for participating nurses through two structured videos (total duration of 4 min), covering clear definition, sources, and types of NIMA. (2) Real‐time observation and adaptive calibration: After training, nurses used the previously published “NIMA observation table” to make real‐time records in 7 consecutive shifts, which included date, shift time, NIMA sources, NIMA types, NIMA outcomes, and an open field (to record the specific description of unclassified events). The purpose of this stage was to enhance the ability of nurses to label NIMA behavior and reduce the definition heterogeneity of subsequent recall stages. (3) Validation of recall window: On the seventh day, a semistructured interview was conducted to ask the key question: “To ensure data accuracy, what do you think is a reasonable time span for retrospective reporting of NIMA occurrence?” The analysis results showed that 80% of nurses (*n* = 8/10) reported that the short‐term retrospective window based on recency effect theory (defined as the last complete shift with reporting time ≤ 12 h) had optimal reliability, which was consistent with the short‐term memory retention pattern derived from the Ebbinghaus forgetting curve [26], supporting the use of the previous shift as the data collection baseline. (4) Data collection and event classification criteria: Based on the above evidence, we adopted the standardized question: “During your last completed shift, how often were you interrupted during medication administration (NIMA)?” Consistent with direct‐observation research, which commonly reports interruption frequency as an hourly rate (number of interruptions divided by observation time in hours) [27], we framed response options as interruptions per hour to facilitate comparison across studies and across shifts of different lengths. Response categories were as follows: rarely or never (< 1 per hour), occasionally (1–3 times per hour), often (4–7 times per hour), and always (≥ 8 times per hour). Because no guideline‐based cutoff exists for NIMA self‐report frequency, we established a conservative cutoff of ≥ 1 interruption per hour (i.e., “occasionally” or higher). This threshold was selected based on empirical benchmarks: While direct‐observation studies often report high interruption rates (ranging from 3 to over 6 times per hour) during medication‐related tasks [28, 29], we utilized a more conservative threshold of ≥ 1/hour to account for potential recall bias and to ensure that the reported events were frequent enough to represent a nontrivial interruption exposure during medication administration. Despite the limitations of potential recall bias, the preexperimental data showed acceptable agreement in positive or negative classification between real‐time logs and retrospective reports, indicating that the design had an acceptable level of confidence in the absence of electronic monitoring tools.

### 2.3. Variable Selection

The initial pool of predictors consisted of 52 items, derived from two distinct sources to ensure both theoretical depth and comprehensive coverage. First, regarding theoretical relevance, the general characteristic variables (parts 1–3) were selected based on a review of relevant literature to capture essential baseline information and potential confounders. The risk factor variables (Part 4) were operationalized from the “Risk Evaluation Index System for NIMA” developed by our team [20]. This system is theoretically grounded in the “Safety Information Flow‐based Accident Causative Conceptual Model,” covering dimensions of human, machine, management, information, resources, and environment. We excluded macrolevel management indicators to ensure the feasibility of individual nurse self‐reporting.

These sections are as follows: (1) Demographic variables (10 items): Department type, gender, age, education level, marital status, number of children, professional title, position, service years, and monthly income. (2) Daily work and life information (7 items): The understanding level of NIMA, actual working hours, resignation intention, sleep duration, sleep problems, physical exercise frequency, and duration. (3) Previous shift variables (2 items): Shift nature and time range. (4) Risk factor variables for NIMA (33 items): (1) Personnel‐related factors: Knowledge, attitude, and behavior levels of NI; unfamiliarity with commonly used drugs in the department; unfamiliarity with commonly used related equipment; handling personal matters; work‐related confusion; circadian rhythm disturbances; physical discomfort; lack of concentration; risk perception of nursing environment; general self‐efficacy; mental workload; job burnout; and needs of doctors, head nurses, colleagues, other hospital staff, patients, their families, and others. (2) Information‐related factors: Incorrect doctor’s orders, incomplete patient identification, and erroneous patient information. (3) Environmental factors: Drug safety culture, department layout, noise pollution, and uncomfortable lighting conditions. (4) Equipment‐related factors: Availability of office equipment, availability of drug‐related instruments, availability of auxiliary facilities, and information system issues. Among these, the levels of knowledge, attitude, and behavior of NI [6, 7], risk perception of nursing environment [30], general self‐efficacy [31], mental workload [32], and job burnout [33] were investigated by using widely used scales.

To ensure methodological rigor and minimize multicollinearity among the 52 candidate predictors, we employed a two‐step screening process. Qualitatively, redundancy among the 33 risk items was previously addressed via a Delphi expert consultation during the scale development phase [20]. Quantitatively, in the current study, univariate analysis was performed prior to multivariate modeling to screen out nonsignificant variables. Finally, collinearity diagnostics (variance inflation factor [VIF] and condition index [CI]) were conducted on the final multivariate model to ensure that no severe multicollinearity existed among the retained predictors. The specific results can be found in Supporting Collinearity analysis.

### 2.4. Sample Size

Sample size estimation was based on the events per variable (EPV) principle, requiring 10–20 events per candidate predictor to ensure model stability and reduce overfitting. Given 52 candidate predictors of the questionnaire on characteristic factors of the prediction model for NIMA, a minimum of 520−1040 NIMA cases were needed. In addition, a NIMA prevalence is about 55% [34], which corresponds to an effective sample size of approximately 946−1891 nurses. Considering a potential 20% rate of invalid responses (e.g., incomplete questionnaires, missing key variables, and logical inconsistencies), we estimated that at least 1183−2364 nurses would need to be recruited to achieve the required number of valid cases for model development and validation.

### 2.5. Data Collection

Eligible nurses were invited to participate via the Wenjuanxing online platform (https://www.wjx.cn/login.aspx). Data were collected through an anonymous, self‐administered questionnaire over a 2‐month period. The survey included an invitation letter outlining key study concepts, objectives, significance, and confidentiality assurances. Before launching the full‐scale survey, a preliminary pilot test was carried out among 30 registered nurses from the departments of hematology and endocrinology. The aim was to evaluate the comprehensibility of the questionnaire items and to detect any technical difficulties related to the use of the online self‐report platform. Subsequently, we contacted the nursing department directors of 12 hospitals and sent them electronic questionnaires. Standardized instructions were provided to explain the significance of institutional participation and to obtain their consent. Following approval, questionnaires were disseminated to qualified nurses through the coordination of nursing directors and managers. Nurses who agreed to participate accessed and completed the survey by clicking the provided link via WeChat/QQ or by scanning a QR code. Before distributing the survey, three investigators (two postgraduate students and one undergraduate student) received training from the principal researcher. They were responsible for overseeing the online questionnaire collection and continuously monitoring data quality and response rates. Any issues encountered were promptly addressed through communication with the respective hospital’s nursing manager.

### 2.6. Model Development and Validation

Nurses were randomly assigned in a 4:1 ratio to the training dataset, which was used to build the prediction model, and the internal dataset, which was used to internally validate the prediction model. Three machine learning algorithms, including logistic regression (LR), decision tree (DT), and Naive Bayes (NB), were employed in R Version 4.4.2 to generate models for predicting NIMA. Each model was optimized by 5 repeats of 10‐fold cross‐validation or tuned to the best parameters. Internal validation was performed using 1000 bootstrap resamples to evaluate the stability of predictive models. The area under the receiver operator characteristic curve (AUC), accuracy, precision, recall, specificity, F1‐score, and G‐mean were used to evaluate the accuracy of prediction models. The reliability of the prediction models was assessed using the Hosmer−Lemeshow test, and calibration curves were plotted to evaluate model fit. The best‐performing predictive model was presented as a nomogram and web‐based calculators, assigning a distinct score to each variable. The aggregate score is then used to estimate the probability of NIMA.

### 2.7. Statistical Analysis

The first step involved composition ratios for categorical variables, and means with standard deviations (SDs) for continuous variables were provided by descriptive analysis. For decreasing bias, we screened all responses for validity before analysis. Questionnaires with a total completion time of less than 30 s or with missing responses on key items, particularly those related to NIMA occurrence, were deemed invalid and excluded from the dataset. Logical consistency checks were also performed to identify and exclude responses with internal contradictions. Subsequently, we conducted univariate analyses of candidate predictors for NIMA among nurses by the *χ* ^2^ test and *t*‐test. The dependent variable was whether it happens or not of NIMA among nurses, and candidate predictor variables were as follows: demographic variables, daily work and life information, previous shift variables, and risk factor variables for NIMA. Given the cross‐sectional nature of the study, sensitivity analyses were not applicable. To address potential confounding, we included all theoretically relevant and statistically significant variables in the multivariable LR model. This approach allows for adjustment of confounding effects by estimating the independent association between each predictor and NIMA while holding other variables constant. Odds ratios (ORs) and 95% confidence intervals (CIs) were calculated to assess the strength of associations. The model development and evaluation were performed using Python software (Version 3.7), while the nomogram was constructed with R software (Version 4.4.2). Additional statistical analyses were conducted using IBM SPSS Statistics Version 26.0. All tests were two‐tailed, with *p* value less than 0.05 considered statistically significant.

## 3. Results

### 3.1. Characteristics of Nurses

We enrolled 4764 registered nurses who met the predefined inclusion and exclusion criteria. However, we excluded six questionnaires (three for regular response patterns and three for incomplete responses), resulting in an effective response rate of 99.87%. Of these, the mean age was 32.68 ± 6.93 years, 4610 nurses (96.9%) were female, 3889 (81.8%) nurses had a bachelor’s degree or above, 3820 (80.3%) nurses had some knowledge of NIMA, and 2480 (52.1%) nurses reported experiencing NIMA during their previous shift (see Table 1 in Supporting Information).

**TABLE 1:** Multivariate logistic regression analysis of predictors for NIMA.

Predictors	*β*	S.E.	Wald	Odds ratio (95% CI)	*p*
*X1 Department type*					
Internal medicine				Rf.	
General surgery	0.058	0.081	0.522	1.060 (0.905, 1.241)	0.470
Obstetrics and pediatrics	0.068	0.104	0.427	1.070 (0.873, 1.313)	0.514
Emergency and critical care	−0.261	0.116	5.033	**0.771 (0.614, 0.968)**	**0.025**
Others	−0.54	0.141	14.742	**0.583 (0.443, 0.768)**	**< 0.001**

*X5 Marital status*					
Single				Rf.	
Currently married	−0.244	0.081	9.133	**0.784 (0.669, 0.918)**	**0.003**

*X10 Monthly income*					
≤ 5000 yuan				Rf.	
5000–10000 yuan	0.390	0.075	26.749	**1.477 (1.274, 1.712)**	**< 0.001**
> 10000 yuan	0.413	0.130	10.087	**1.511 (1.171, 1.949)**	**0.001**

*X13 Resignation intention*					
Never				Rf.	
Rarely	0.201	0.097	4.32	**1.222 (1.012, 1.477)**	**0.038**
Sometimes	0.632	0.092	47.711	**1.882 (1.573, 2.251)**	**< 0.001**
Often	1.047	0.164	40.875	**2.849 (2.067, 3.927)**	**< 0.001**
Always	0.618	0.301	4.219	**1.855 (1.029, 3.347)**	**0.040**

*X16 Physical exercise*					
Never				Rf.	
1–2 times/week	0.226	0.071	9.978	**1.253 (1.089, 1.441)**	**0.002**
> 3 times/week	0.273	0.162	2.851	**1.314 (0.957, 1.803)**	**0.091**

*X18 Shift type*					
Charge nurse shift				Rf.	
Primary nursing duty	0.369	0.117	9.898	**1.447 (1.149, 1.821)**	**0.002**
Support nurse shift	0.036	0.171	0.043	1.036 (0.741, 1.449)	0.836
Clinical quality oversight	0.196	0.195	1.018	1.217 (0.831, 1.782)	0.313
Other specialty roles	0.313	0.124	6.407	**1.368 (1.073, 1.744)**	**0.011**

*X19 Shift time range*					
AM shift				Rf.	
PM shift	−0.001	0.127	0	0.999 (0.778.281)	0.991
Night shift	−0.308	0.113	7.414	**0.735 (0.589, 0.917)**	**0.006**
Day duty	0.041	0.097	0.174	1.041 (0.861, 1.260)	0.676
Others	−0.237	0.133	3.207	**0.789 (0.608, 1.023)**	**0.073**
X22 The behavior level of NI	−0.043	0.007	44.035	**0.958 (0.945, 0.970)**	**< 0.001**
X31 General self‐efficacy level	0.007	0.002	13.752	**1.007 (1.004, 1.011)**	**< 0.001**

*X34 Needs of doctors*					
No				Rf.	
Yes	0.565	0.096	34.559	**1.760 (1.458, 2.125)**	**< 0.001**

*X35 Needs of head nurse*					
No				Rf.	
Yes	−0.374	0.104	13.048	**0.688 (0.561, 0.843)**	**< 0.001**

*X36 Needs of colleagues*					
No				Rf.	
Yes	0.312	0.093	11.333	**1.366 (1.139, 1.639)**	**0.001**

*X37 Needs of other hospital staff*					
No				Rf.	
Yes	0.275	0.102	7.229	**1.316 (1.077, 1.608)**	**0.007**

*X38 Needs of patients*					
No				Rf.	
Yes	0.672	0.09	55.517	**1.958 (1.641, 2.336)**	**< 0.001**

*X39 Needs of patients’ family members*					
No				Rf.	
Yes	0.369	0.093	15.826	**1.446 (1.206, 1.734)**	**< 0.001**

*X41 Incorrect doctor’s orders*					
No				Rf.	
Yes	−0.335	0.112	8.923	**0.715 (0.574, 0.891)**	**0.003**

*X45 Unreasonable functional zoning of departments*					
No				Rf.	
Yes	0.167	0.077	4.686	**1.182 (1.016, 1.375)**	**0.030**
X48 Insufficient medication supplies					
No				Rf.	
Yes	0.179	0.076	5.520	**1.197 (1.030, 1.390)**	**0.019**

*Note:* Bold values indicate statistical significance (*p* < 0.05). NIMA: nursing interruptions during medication administration.

### 3.2. Feature Selection

As shown in Table 1 in Supporting Information, after univariate analyses, 44 potential predictive factors were statistically significant according to the criterion of *p* ≤ 0.10. No significant differences were observed between nurse characteristics in the training and internal test sets. Subsequently, an LR analysis was conducted utilizing the forward stepwise selection method (likelihood ratio test) to refine the variables, with significance levels set at 0.05 for entry and 0.10 for removal. The results showed that 18 selected features were finally included in the predictive models (Table 1), including department type, marital status, monthly income, resignation intention, physical exercise, shift type, shift time range, the behavior level of NI, the level of general self‐efficacy, needs from doctors, head nurses, colleagues, other hospital staff, patients, and patients’ family members, incorrect doctor’s orders, unreasonable functional zoning of departments, and insufficient medication supplies.

### 3.3. Model Performance and Comparison

Based on the 18 variables selected, we constructed NIMA risk prediction models using LR, DT, and NB algorithms. As summarized in Table 2, the AUCs across models ranged from 0.679 to 0.770 in the training and internal test sets, with LR showing the highest AUC. The receiver operating characteristic (ROC) curves for the training and internal validation sets are shown in Figures 1(a) and 1(b), respectively. To further evaluate model performance, confusion matrix analyses were performed. For example, the LR model indicators were comprehensively evaluated, achieving an accuracy of 0.709 (95% CI: 0.694–0.723) in the test set. Other performance metrics, including precision, sensitivity, specificity, F1‐score, and G‐mean, are detailed in Table 2. The confusion matrices for all models are presented in Supporting Figure S1.

**TABLE 2:** Performance metrics of NIMA risk prediction models in the training and internal test sets.

Model	AUC (95% CI)	Accuracy (95% CI)	Precision	Sensitivity	Specificity	*F*1‐score	*G*‐mean
*Training set*							
LR model	0.770 (0.755,0.785)^∗∗^	0.709 (0.694, 0.723)^∗∗^	0.721	0.722	0.694	0.721	0.708
DT model	0.700 (0.685, 0.715)^∗∗^	0.695 (0.681, 0.710)^∗∗^	0.742	0.696	0.695	0.718	0.696
NB model	0.754 (0.734,0.770)^∗∗^	0.700 (0.685, 0.715)^∗∗^	0.665	0.736	0.668	0.699	0.701

*Internal test set*							
LR model	0.748 (0.717, 0.779)^∗∗^	0.694 (0.664, 0.724)^∗∗^	0.716	0.697	0.691	0.706	0.694
DT model	0.679 (0.648, 0.709)^∗∗^	0.672 (0.641, 0.702)^∗∗^	0.724	0.667	0.679	0.694	0.673
NB model	0.745 (0.714,0.777)^∗∗^	0.699 (0.668, 0.728)^∗∗^	0.654	0.731	0.671	0.690	0.700

Abbreviations: DT = decision tree; LR = logistic regression; NB = Naive **B**ayes.

^∗∗^ *p* < 0.001.

FIGURE 1Receiver operating characteristic (ROC) curves of the three machine learning models. (a) ROC curves in the training set. (b) ROC curves in the internal test set.

**Figure:**
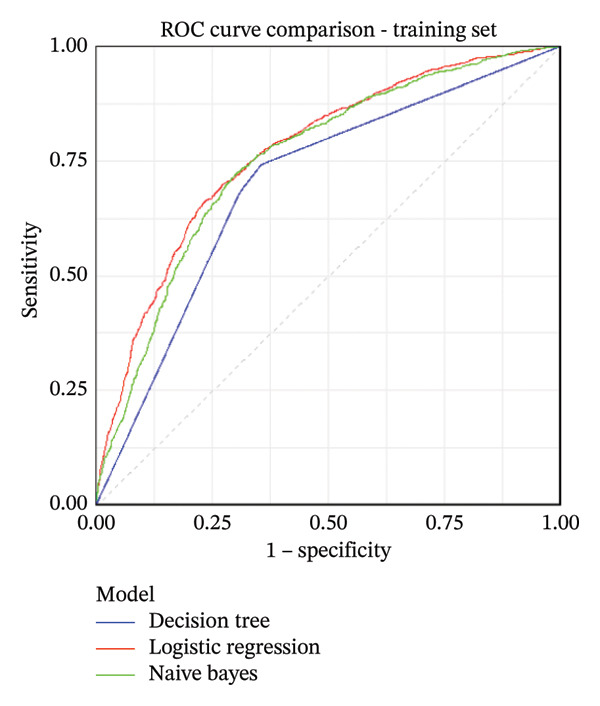
(a)

**Figure:**
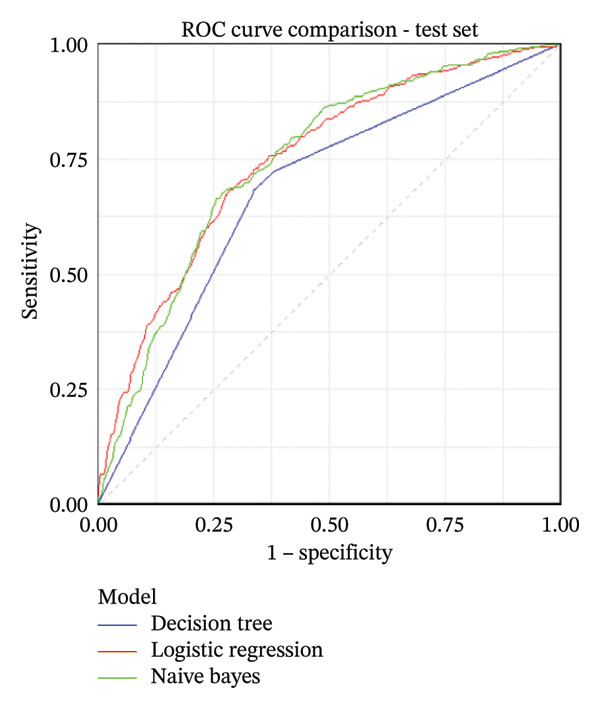
(b)

As shown in Tables 2 and 3, in the training set, the AUCs (95% CI) of the LR model (0.770 [0.755,0.785]) and NB model (0.754 [0.734,0.770]) were significantly higher than those of the DT model (0.700 [0.685, 0.715]) by the DeLong test (*p* < 0.001). In addition, there was a significant difference in AUC between the LR model and the NB model, with the LR model having a higher AUC (*p* < 0.001). In the internal test set, the AUCs (95% CI) of the LR model (0.748 [0.717, 0.779]) and the NB model (0.745 [0.714, 0.777]) were significantly higher than those of the DT model (0.679 [0.648, 0.709]) by the DeLong test (*p* < 0.001). However, the AUC difference between the LR model and the NB model was not significant (*p* = 0.680); although the AUC of the LR model was slightly higher, the difference was marginal. Since the LR model had the highest AUC of 0.748 (0.717, 0.779), we used the Hosmer−Lemeshow test to investigate the LR model. The results of the training set were *χ* ^2^ = 7.475, *p* = 0.486, and results of the internal test set were *χ* ^2^ = 7.062, *p* = 0.530, indicating that the LR model had a good fitting effect and the predicted results were in high consistency with the actual situation.

**TABLE 3:** Comparison of AUC values in training set and internal test set (DeLong test).

Model A vs. Model B		*Z*	95% CI	*p*
*Training set*				
LR model	DT model	13.546	(0.060, 0.081)	**<** **0.001**
LR model	NB model	4.696	(0.009, 0.023)	**<** **0.001**
Decision tree model	NB model	−10.439	(−0.065, −0.044)	**<** **0.001**

*Internal test set*				
LR model	DT model	6.447	(0.048, 0.091)	**<** **0.001**
LR model	NB model	0.412	(−0.012 0.017)	0.680
Decision tree model	NB model	−6.204	(−0.088, −0.046)	**<** **0.001**

*Note:* Bold text indicates statistically significant results (*p* < 0.05).

Abbreviations: DT = decision tree; LR = logistic regression; NB = Naive Bayes.

**FIGURE 2:**
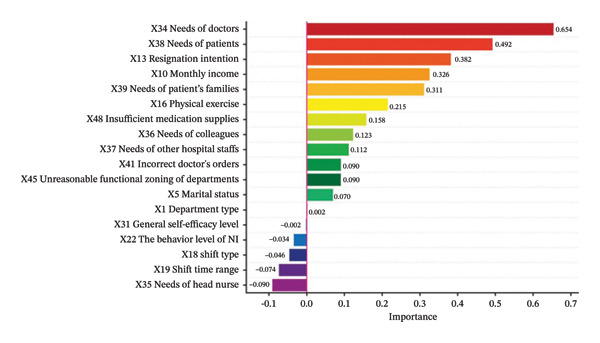
Histogram of feature importance score for the LR model.

Then, in order to explore the importance of each feature in the best prediction model, we applied the feature importance score (FIS) to interpret the LR model globally. The higher the FIS score, the greater the influence of the feature on the model prediction. Figure 2 shows the FIS and ranking of the LR model. The ranking showed the importance of each feature in predicting the occurrence of NIMA, that is, the needs of doctors (0.654) contributed the most to the predicted outcome, while department type (0.002) and the general self‐efficacy level (−0.002) of nurses had relatively little influence. Furthermore, the following features were associated with an increased probability of NIMA: needs of doctors, patients, patients’ family members, colleagues, other hospital staff, resignation intention, monthly income, physical exercise, insufficient medication supplies, doctors’ orders, unreasonable functional zoning of departments, marital status, and department type. Conversely, the following features were associated with a reduced probability of NIMA: general self‐efficacy level, the behavior level of NI, the shift type, the shift time range, and the needs of head nurses.

### 3.4. Nomogram

**FIGURE 3:**
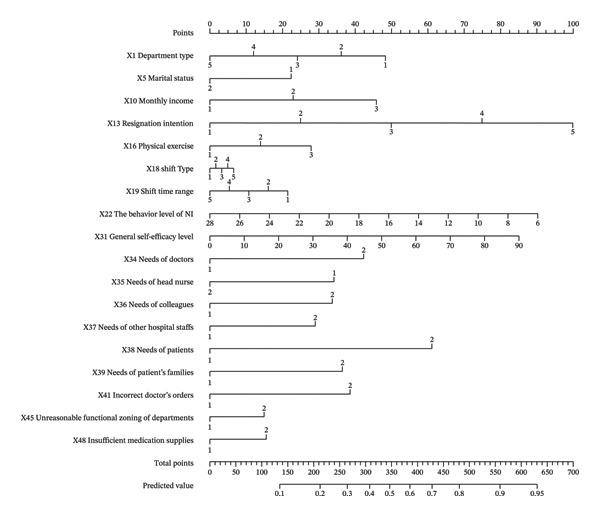
Nomogram predicting the probability of NIMA. Notes: X1‐department type (1‐internal medicine; 2‐general surgery; 3‐obstetrics and pediatrics; 4‐emergency and critical care; 5‐others); X5‐marital status (1‐single; 2 currently married); X10 monthly income (1‐≤ 5000 yuan; 2–5000‐10000 yuan; 3‐> 10000 yuan); X13‐resignation intention (1‐never; 2‐rarely; 3‐sometimes; 4‐often; 5‐always); X16‐physical exercise (1‐never; 2‐1‐2 time/week; 3‐> 3 time/week); X18‐shift‐type (1‐charge nurse shift; 2‐primary nursing duty; 3‐support nurse shift; 4‐clinical quality oversight; 5‐other specialty roles); X19‐shift time range (1‐AM shift; 2‐PM shift; 3‐night shift; 4 day duty; 5‐others); X22‐the behavior level of NI; X31‐general self‐efficacy level; X34‐needs of doctors (1‐no; 2‐yes); X35‐needs of head nurse (1‐no; 2‐yes); X36‐needs of colleagues (1‐no; 2‐yes); X37‐needs of other hospital staff (1‐no; 2‐yes); X38‐needs of patients (1‐no; 2‐yes); X39‐needs of patients’ family members (1‐no; 2‐yes); X41‐incorrect doctors’ orders (1‐no; 2‐yes); X45‐unreasonable functional zoning of departments (1‐no; 2‐yes); X48‐insufficient medication supplies (1‐no; 2‐yes).

Most machine learning models lack interpretability and are not easily visualized. Given its satisfactory performance, the LR model was converted into a nomogram to facilitate clinical application (Figure 3), with each predictor assigned a point value to estimate the NIMA risk. The construction approach was informed by prior work in predictive modeling among nurses [25]. To demonstrate the clinical utility of the nomogram, we analyzed a hypothetical case of a nurse with a high‐risk profile (e.g., “always” having resignation intention, frequent interruptions from various sources, and high monthly income). The detailed scoring process for this case is provided in Supplementary_Example 1. By aggregating the individual scores for each variable, the total score for this nurse amounted to 696.25 points. Aligning this total score vertically with the probability scale on the nomogram indicated a > 99% probability of NIMA occurrence.

While the 18‐variable nomogram provides a comprehensive risk assessment, collecting 18 variables may be time‐consuming for routine bedside application. To address this, we further screened the 18 predictors based on the multivariate LR results, specifically prioritizing variables with high ORs, significant *p* values, and strong clinical relevance. Ultimately, we selected 5 core variables, including monthly income, resignation intention, needs of doctors, patients, and patients’ family members, to develop a simplified practical model. The simplified 5‐variable model maintained robust predictive performance (detailed in Supporting Table 4). To maximize clinical convenience, we translated these models into interactive web‐based calculators. The simplified 5‐variable practical version, recommended for rapid bedside assessment, is freely accessible at https://nursing-research.shinyapps.io/NIMA_Predictor2/. In addition, the full 18‐variable version, suitable for institutions with automated electronic health record integration, is available at https://nursing-research.shinyapps.io/NIMA_Predictor/(screenshots of both web applications are provided in Supporting Figure S2).

## 4. Discussion

This study is the first large‐scale and systematic investigation of the factors associated with NIMA among Chinese nurses. We initially considered 52 candidate predictors (demographic, work‐related, and risk‐factor variables) to comprehensively capture potential determinants of NIMA. After feature selection, 18 predictors were retained and used to develop and internally validate three machine learning models (LR, NB, and DT). Comparative analysis showed that LR achieved the most balanced and superior performance in terms of discrimination, calibration, and overall classification metrics. We further developed a nomogram based on the LR model, offering individualized NIMA risk estimates and serving as a practical, interpretable decision‐support tool to assist nursing managers in identifying high‐risk nurses and optimizing medication safety strategies.

### 4.1. Influencing Factors of NIMA Among Nurses

In this multicenter cross‐sectional study, the prevalence of NIMA among nurses was 52.1%. This finding showed a similar prevalence of NIMA with a randomized controlled study among nurses conducted in Australia [34], which reported a prevalence rate of 55.8%, and did not investigate the related influencing factors of NIMA. In this study, we comprehensively and systematically reflected on the risk factors of NIMA, including personnel‐related factors and department‐related factors.

#### 4.1.1. Personnel‐Related Factors

##### 4.1.1.1. The Needs of Patients, Their Families, Doctors, Nurse Colleagues, and Other Staffs

Interpersonal demands from patients, families, doctors, and colleagues were the most salient predictors of NIMA, highlighting the complex social dynamics embedded in clinical workflows. Consistent with prior studies across pediatric, respiratory, and ICU settings [12, 14, 27, 35], these interruptions often stemmed from nontherapeutic queries, such as referral procedures, insurance matters, or discharge pressures, that divert nurses’ attention during high‐risk medication tasks [11]. Notably, over 80% of interruptions have been attributed to patients’ family members, environmental noise, and physicians [12], and up to 87.9% involve tasks unrelated to pharmacologic safety [34], underscoring the misalignment between interruption sources and clinical priorities. These frequent, cognitively taxing disruptions compromise vigilance and heighten medication error risk, especially in settings with understaffing and limited support resources [7]. Addressing such systemic vulnerability calls for multifaceted strategies, including establishing no‐interruption zones [36], implementing task‐switching, and interruption management training through simulation [37]. Our findings further support the need for structural reforms that reduce low‐value interruptions while preserving essential interdisciplinary communication [12, 34, 38, 39].

##### 4.1.1.2. Nurses’ Personal Factors

Resignation intention, monthly income, and general self‐efficacy were identified as key risk factors for NIMA. Nurses with frequent resignation intention were significantly more likely to experience interruptions, consistent with prior findings that job burnout and fatigue impair attention, increase cognitive load, and elevate error risk [40, 41]. Higher income, often correlated with seniority and role‐related demands, was associated with increased NIMA risk. One possible hypothesis is that higher income may co‐occur with unmeasured motivational or attentional factors that could increase susceptibility to interruptions; however, these factors were not directly assessed in our study. Therefore, this association should be interpreted cautiously. Notably, general self‐efficacy was positively associated with NIMA risk. Although self‐efficacy is generally considered beneficial [42], individuals with high self‐belief may overestimate their multitasking ability and underestimate task complexity, especially under limited attention resources, which is in line with the multiple resource theory [43]. This may lead to riskier behavior and cognitive overload. However, prior evidence remains mixed; one randomized trial found no difference in medication error rates between students with high and low self‐efficacy [44], underscoring the gap between perceived competence and actual performance [45]. These findings emphasize the importance of psychological and behavioral factors in shaping nurses’ vulnerability to NIMA. In addition to technical training, targeted interventions, such as simulation‐based learning tailored to individual characteristics (e.g., age, position, and marital status) [6, 7] and systematic monitoring of burnout and turnover intention, may enhance coping ability, reduce distraction, and improve medication safety outcomes.

#### 4.1.2. Department‐Related Factors

In addition, our study further found that insufficient medication supplies and unreasonable functional zoning of departments increased the risk of medication‐related interruptions. These issues may cause delays as nurses search for medications or navigate inefficient workspaces and were also error‐provoking conditions influencing administration errors [46]. In addition, about shift type, primary nurses had a lower risk of NIMA compared to charge nurses, possibly due to differences in their responsibilities and work pace. Similarly, night‐shift nurses experienced fewer NIMA than those on day shifts, which could be attributed to fewer disturbances, reduced patient activity, and a more stable workflow during nighttime hours. Previous observation study also found that NIs were more prevalent during handover from the day to evening shift [47]. However, in a cohort study involving 257 nurses and 3308 patients in a pediatric intensive care unit, night‐shift nurses interrupted by phone calls had a significantly increased risk of medication errors (OR = 1.35, 95% CI: 1.16–1.57, *p* < 0.001) [5]. These findings suggest the NIMA was influenced by multiple factors, including the work environment and shift arrangements. Therefore, nursing managers should optimize resource allocation and adjust shift task distribution to minimize interruptions and enhance medication safety.

### 4.2. Development and Evaluation of Predictive Models for NIMA Among Nurses Based on Machine Learning Algorithms

Currently, only one predictive model has been developed to assess the risk of NIs, with an AUC of 0.891. However, it was limited to the context of operating room checklists and relied solely on LR [48]. In contrast, this study is the earliest to develop and evaluate applicable and moderately individualized models for predicting NIMA. All three models demonstrated acceptable predictive performance in the training and internal test sets. Overall, the LR model showed the most consistent performance across evaluation domains and provided the best trade‐off between predictive performance and interpretability, which is consistent with prior work [49]. It is plausible that LR performed competitively because many predictors were categorical or ordinal, and their effects may be largely additive and approximately linearly separable after feature selection. In contrast, DT can be more sensitive to measurement noise and correlated inputs, leading to less stable splits, while NB may be constrained when its conditional independence assumption is violated by interrelated interruption sources and work‐related factors. These explanations are hypotheses rather than mechanisms directly tested in this study.

Based on the comprehensive 18‐variable LR model, a nomogram was initially developed to map the full risk profile of NIMA. However, we acknowledge that manually collecting 18 variables is highly impractical for routine, fast‐paced clinical workflows. To bridge the gap between theoretical modeling and real‐world implementation, we distilled the model into a 5‐variable practical tool and deployed both models as interactive web‐based applications. The simplified 5‐variable calculator serves as the primary, user‐friendly tool for daily bedside assessment. In clinical practice, nursing managers can easily integrate this simplified calculator with existing observation tools to support proactive risk stratification without adding significant workload. For example, even if a nurse has not experienced NIMA during a recent observation period, the calculator can identify their underlying risk, facilitating targeted supervision and preventive interventions rather than relying solely on past events. Meanwhile, the full 18‐variable web version remains available as a supporting tool, specifically suited for future research or institutions with automated Electronic Health Record integration capabilities. Ultimately, this tiered approach directly addresses the need for both scientific rigor and accessible clinical risk assessment.

### 4.3. Limitation

Despite its strengths, this study has several limitations. First, regarding model validation, although we employed bootstrapping techniques to ensure robust internal validity, the absence of external validation remains a primary limitation. Since all data were derived from a limited number of hospitals in central China, the generalizability of the model to other regions or healthcare settings remains uncertain. Future research should prioritize collecting independent external datasets and examining feasibility issues such as additional workload, training needs, and integration into existing digital systems (e.g., HIS/EMR) to further test the model’s performance and real‐world utility. Second, reliance on self‐reporting introduces potential recall bias. Given the high‐frequency and fragmented nature of NIMA (often occurring multiple times per hour), relying solely on subjective recall may lead to underestimation or selective forgetting, particularly for low‐severity interruptions. Third, while the model included a comprehensive set of predictors, certain potentially influential factors were not examined. Variables such as situational awareness, patient safety attitudes, and specific details regarding medication administration (e.g., drug types and procedures) were not considered in this study and warrant investigation in future work. Finally, although the timing of NIMA is important for understanding its frequency and patterns, it was not assessed in this study. We intend to explore this in future investigations.

## 5. Conclusion

The study represents the first large‐scale, systematic investigation of the factors associated with NIMA among Chinese nurses. Based on this comprehensive evaluation, we developed and validated three machine learning–based risk prediction models (LR, NB, and DT), enabling early identification of nurses at high risk for NIMA. These models serve as proactive decision‐support tools to enhance the precision of risk assessment and facilitate timely, targeted interventions. However, before routine adoption in nursing management workflows, external validation in independent and prospective cohorts and implementation feasibility evaluation (e.g., workload implications, staff training, and digital integration) are warranted. With such further validation and implementation work, integrating these tools into clinical workflows may help improve patient medication safety and support data‐driven nursing management.

## Author Contributions

Xiaoqian Dong: writing−review and editing, writing−original draft, software, methodology, data curation, and conceptualization. Siqing Ding: writing−review and editing, methodology, data curation, and conceptualization. Sha Wang: writing−review and editing, methodology, and investigation. Min Liu: software, methodology, conceptualization, and investigation. Gang Gan: software, methodology, conceptualization, and investigation. Huan Cao: software, methodology, data curation, and conceptualization. Xingxing Wang: software, methodology, data curation, and conceptualization. Nandan Chen: software, methodology, data curation, and investigation. Bangdi Tan: software, methodology, data curation, and conceptualization. Jianfei Xie: writing−review and editing, writing−original draft, methodology, investigation, conceptualization, funding acquisition, and project administration. H. F. Peter Ng: software, writing−review and editing, methodology, investigation, and conceptualization.

## Funding

This work was supported by the Wisdom Accumulation and Talent Cultivation Project of the Third Xiangya Hospital of Central South University (Grant no. BJ202205), the Hunan Natural Science Foundation‐Distinguished Young Scholars (Grant no. 2022JJ10098), and the Graduate Independent Exploration and Innovation project of Central South University (Grant no. 820).

## Disclosure

All authors have read and agreed to the published version of the manuscript. Guidelines and Standards Statement: This manuscript follows the checklist items of STROBE Statement about cross‐sectional studies.

## Ethics Statement

Institutional Review Board Statement: Ethical approval was obtained from the review boards of all participating hospitals. Participants provided informed consent prior to enrollment, with assurances of confidentiality upheld throughout the study. All collected data were handled with strict confidentiality.

## Consent

Please see the Ethics Statement.

## Conflicts of Interest

The authors declare no conflicts of interest.

## Supporting Information

Supporting 1. Supporting Table 1 presents the univariate analyses of 52 candidate predictors associated with NIMA among 4758 nurses. Supporting Table 2 compares the characteristics between the training and internal tests. Due to the large number of variables, these tables were provided separately as supplementary files. In addition, Supporting Table 3 details the collinearity diagnostics (tolerance and VIF) to rule out severe multicollinearity among the variables. Supporting Figure S1 illustrates the confusion matrices for the LR, DT, and NB models across both the training and internal test sets, providing a visual breakdown of classification performance. Finally, Supporting Example 1 presents a hypothetical case study to demonstrate the practical application and point calculation process of the risk prediction model. Supporting Table 4 presents the performance metrics of the simplified 5‐variable LR model. Supporting Figure S2 displays the interfaces of the two web‐based calculators developed for clinical application.

## Supporting information


**Supporting Information** Additional supporting information can be found online in the Supporting Information section.

## Data Availability

Data are available on request from the authors. The data that support the findings of this study are available from the corresponding author upon reasonable request.

## References

[1] Patient Safety, 2023, https://www.who.int/news-room/fact-sheets/detail/patient-safety.

[2] Medication Without Harm, 2021, https://www.who.int/initiatives/medication-without-harm.

[3] Massó Guijarro P. , Aranaz Andrés J. M. , Mira J. J. , Perdiguero E. , and Aibar C. , Adverse Events in Hospitals: The Patient’s Point of View, Quality and Safety in Health Care. (2010) 19, no. 2, 144–147, 10.1136/qshc.2007.025585, 2-s2.0-77950446520. 20351163

[4] Westbrook J. I. , Duffield C. , Li L. , and Creswick N. J. , How Much Time do Nurses Have for Patients? A Longitudinal Study Quantifying Hospital Nurses’ Patterns of Task Time Distribution and Interactions with Health Professionals, BMC Health Services Research. (2011) 11, no. 1, 10.1186/1472-6963-11-319, 2-s2.0-82055181582. PMC3238335 22111656

[5] Bonafide C. P. , Miller J. M. , Localio A. R. et al., Association Between Mobile Telephone Interruptions and Medication Administration Errors in a Pediatric Intensive Care Unit, JAMA Pediatrics. (2020) 174, no. 2, 162–169, 10.1001/jamapediatrics.2019.5001. 31860017 PMC6990809

[6] Xie J. , Sun Q. , Tang S. et al., Knowledge, Attitude and Practice Regarding Nursing Interruptions Among Chinese Nurses: A Nationwide cross-sectional Survey, International Journal of Nursing Science. (2020) 7, no. 1, 66–73, 10.1016/j.ijnss.2019.12.004. PMC7031111 32099862

[7] Li J. , Wang S. , Wu X. et al., Factors to Predict the Knowledge, Attitude and Practice of Nursing Interruptions Among Nurses: A Nationwide Cross-Sectional Survey, Nurse Education in Practice. (2022) 64, 10.1016/j.nepr.2022.103428. 35970094

[8] Hayes C. , Jackson D. , Davidson P. M. , and Power T. , Medication Errors in Hospitals: A Literature Review of Disruptions to Nursing Practice During Medication Administration, Journal of Clinical Nursing. (2015) 24, no. 22, 3063–3076, 10.1111/jocn.12944, 2-s2.0-84943663493. 26255621

[9] Berg L. M. , Källberg A. S. , Göransson K. E. , östergren J. , Florin J. , and Ehrenberg A. , Interruptions in Emergency Department Work: an Observational and Interview Study, BMJ Quality and Safety. (2013) 22, no. 8, 656–663, 10.1136/bmjqs-2013-001967, 2-s2.0-84880888720. 23584208

[10] Zhao J. W. , Zhang X. L. , Lan Q. L. et al., Interruptions Experienced by Nurses During Pediatric Medication Administration in China: An Observational Study, Journal for Specialists in Pediatric Nursing. (2019) 24, no. 4, 10.1111/jspn.12265, 2-s2.0-85073088404. 31332933

[11] Lin T. , Feng X. , Gao Y. et al., Nursing Interruptions in Emergency Room in China: An Observational Study, Journal of Nursing Management. (2021) 29, no. 7, 2189–2198, 10.1111/jonm.13372. 33993569

[12] Wang W. , Jin L. , Zhao X. , Li Z. , and Han W. , Current Status and Influencing Factors of Nursing Interruption Events, American Journal of Managed Care. (2021) 27, no. 6, e188–e194, 10.37765/ajmc.2021.88667. 34156222

[13] Kwon Y. E. , Kim M. , and Choi S. , Degree of Interruptions Experienced by Emergency Department Nurses and Interruption Related Factors, International Emergency Nursing. (2021) 58, 10.1016/j.ienj.2021.101036. 34332454

[14] Schutijser B. , Klopotowska J. E. , Jongerden I. P. , Spreeuwenberg P. , De Bruijne M. C. , and Wagner C. , Interruptions During Intravenous Medication Administration: A Multicentre Observational Study, Journal of Advanced Nursing. (2019) 75, no. 3, 555–562, 10.1111/jan.13880, 2-s2.0-85056859510. 30334590

[15] Wilkes S. M. , Barber L. K. , and Rogers A. P. , Development and Validation of the Workplace Interruptions Measure, Stress and Health. (2018) 34, no. 1, 102–114, 10.1002/smi.2765, 2-s2.0-85021230386. 28639737

[16] Yu E. and Lee E. , Development and Validation of a Nursing Work Interruption Scale, International Journal of Environmental Research and Public Health. (2022) 19, no. 20, 10.3390/ijerph192013487. PMC9602459 36294067

[17] Eid T. , Machudo S. , and Eid R. , Interruptions During Medication Work in a Saudi Arabian Hospital: An Observational and Interview Study of Nurses, Journal of Nursing Scholarship. (2022) 54, no. 5, 639–647, 10.1111/jnu.12765. 35064618

[18] Ma J. , Bai Y. , Xie D. , and Yang G. , Factors Influencing the Interruption of Nursing Document Writing in the Intensive Care Unit: A Cross-Sectional Survey, Journal of Multidisciplinary Healthcare. (2023) 16, 419–427, 10.2147/JMDH.S394817. 36820218 PMC9938661

[19] Lang H. , Chao W. , and Jian M. , An Accident-Causing Model Under the Perspective of Safety Information Flow, Management Review. (2020) 32, 274–285.

[20] Xiaoqian D. , Siqing D. , Huan C. et al., Construction and Application of Risk Evaluation Index System of Nursing Interruptions in Medication Process, Chinese Journal of Critical Care Nursing. (2024) 5, no. 012, 731–736, 10.3761/j.issn.2096-7446.2024.

[21] Hur S. , Min J. Y. , Yoo J. et al., Development and Validation of Unplanned Extubation Prediction Models Using Intensive Care Unit Data: Retrospective, Comparative, Machine Learning Study, Journal of Medical Internet Research. (2021) 23, no. 8, 10.2196/23508. PMC8387891 34382940

[22] Ma G. , Chen S. , Peng S. et al., Construction and Validation of a Nomogram Prediction Model for the Catheter-Related Thrombosis Risk of Central Venous Access Devices in Patients with Cancer: A Prospective Machine Learning Study, Journal of Thrombosis and Thrombolysis. (2025) 58, no. 2, 220–231, 10.1007/s11239-024-03045-3. 39363143

[23] Choi R. Y. , Coyner A. S. , Kalpathy-Cramer J. , Chiang M. F. , and Campbell J. P. , Introduction to Machine Learning, Neural Networks, and Deep Learning, Translational Vision Science & Technology. (2020) 9, no. 2, 10.1167/tvst.9.2.14. PMC7347027 32704420

[24] Lei L. , Zhang S. , Yang L. et al., Machine Learning-based Prediction of Delirium 24 H After Pediatric Intensive Care Unit Admission in Critically Ill Children: a Prospective Cohort Study, International Journal of Nursing Studies. (2023) 146, 10.1016/j.ijnurstu.2023.104565. 37542959

[25] Xie W. , Liu M. , Okoli C. T. C. et al., Construction and Evaluation of a Predictive Model for Compassion Fatigue Among Emergency Department Nurses: a Cross-Sectional Study, International Journal of Nursing Studies. (2023) 148, 10.1016/j.ijnurstu.2023.104613. 37839306

[26] Wollstein Y. and Jabbour N. , Spaced Effect Learning and Blunting the Forgetfulness Curve, Ear, Nose, & Throat Journal. (2022) 101, no. 9, 42S–46S, 10.1177/01455613231163726. 36880338

[27] Schroers G. , Characteristics of Interruptions During Medication Administration: an Integrative Review of Direct Observational Studies, Journal of Clinical Nursing. (2018) 27, no. 20, 3462–3471, 10.1111/jocn.14587, 2-s2.0-85050528094. 29945303

[28] Schroers G. , Tell D. , and O’Rourke J. , Association of External Interruptions with Increased Medication Administration Duration and Self-Interruptions: A Direct Observational Study: Empirical Research Quantitative, Journal of Advanced Nursing. (2023) 79, no. 11, 4339–4347, 10.1111/jan.15674. 37070669

[29] Westbrook J. I. , Woods A. , Rob M. I. , Dunsmuir W. T. , and Day R. O. , Association of Interruptions With an Increased Risk and Severity of Medication Administration Errors, Archives of Internal Medicine. (2010) 170, no. 8, 683–690, 10.1001/archinternmed.2010.65, 2-s2.0-77951664144. 20421552

[30] Xinwei Z. , Study on Questionnaire Construction of Risk Perception and Influence Factors Analysis for Nurse, 2016, Fourth Military Medical University.

[31] Schwarzer R. , Jerusalem M. , and Weinman J. , Generalized Self-Efficacy Scale, 1995, 10.1037/t00393-000.

[32] Hart S. G. and Staveland L. E. , Hancock P. A. and Meshkati N. , Development of NASA-TLX (Task Load Index): Results of Empirical and Theoretical Research, 1988, Advances in Psychology North-Holland, 139–183.

[33] Maslach C. , Job Burnout: New Directions in Research and Intervention, Current Directions in Psychological Science. (2003) 12, no. 5, 189–192, 10.1111/1467-8721.01258, 2-s2.0-0142022808.

[34] Westbrook J. I. , Li L. , Hooper T. D. , Raban M. Z. , Middleton S. , and Lehnbom E. C. , Effectiveness of a ’Do Not Interrupt’ Bundled Intervention to Reduce Interruptions During Medication Administration: A Cluster Randomised Controlled Feasibility Study, BMJ Quality and Safety. (2017) 26, no. 9, 734–742, 10.1136/bmjqs-2016-006123, 2-s2.0-85029952441. PMC5574391 28232390

[35] Colligan L. and Bass E. J. , Interruption Handling Strategies During Paediatric Medication Administration, BMJ Quality and Safety. (2012) 21, no. 11, 912–917, 10.1136/bmjqs-2011-000292, 2-s2.0-84871932953. 22791692

[36] Anthony K. , Wiencek C. , Bauer C. , Daly B. , and Anthony M. K. , No Interruptions Please: Impact of a No Interruption Zone on Medication Safety in Intensive Care Units, Critical Care Nurse. (2010) 30, no. 3, 21–29, 10.4037/ccn2010473, 2-s2.0-77956697132. 20067939

[37] Schroers G. , Pfieffer J. , Andersen B. , and O’Rourke J. , An Interruption Management Education Bundle: Feasibility Testing with Nursing Students, Nurse Educator. (2024) 49, no. 4, 189–194, 10.1097/NNE.0000000000001583. 38086173

[38] Nowell L. , Ferreira C. , Dhingra S. , Davidson K. , Morgan P. , and Thomas C. , Students and Simulation Facilitators’ Experiences and Perceptions of a Distraction and Interruption Simulation: A Mixed-Methods Study, Nurse Education Today. (2023) 120, 10.1016/j.nedt.2022.105634. 36399861

[39] Vital C. J. and Nathanson B. H. , Effects of the Interruption Management Strategy “Stay S.A.F.E.” During Medication Administration, Rehabilitation Nursing. (2023) 48, no. 2, 65–74, 10.1097/RNJ.0000000000000404. 36792960

[40] Kwon C. Y. , Lee B. , Kwon O. J. , Kim M. S. , Sim K. L. , and Choi Y. H. , Emotional Labor, Burnout, Medical Error, and Turnover Intention Among South Korean Nursing Staff in a University Hospital Setting, International Journal of Environmental Research and Public Health. (2021) 18, no. 19, 10.3390/ijerph181910111. PMC8507784 34639412

[41] Ran L. , Chen X. , Peng S. , Zheng F. , Tan X. , and Duan R. , Job Burnout and Turnover Intention Among Chinese Primary Healthcare Staff: the Mediating Effect of Satisfaction, BMJ Open. (2020) 10, 10.1136/bmjopen-2019-036702. PMC7542935 33033013

[42] Bandura A. , Self-Efficacy: toward a Unifying Theory of Behavioral Change, Psychology Review. (1977) 84, no. 2, 191–215, 10.1037//0033-295x.84.2.191. 847061

[43] Basil M. D. , Seel N. M. , Multiple Resource Theory, Encyclopedia of the Sciences of Learning, 2012, Springer US, Boston, MA, 2384–2385, 10.1007/978-1-4419-1428-6.

[44] Chan R. , Booth R. , Strudwick G. , and Sinclair B. , Nursing Students’ Perceived Self-Efficacy and the Generation of Medication Errors with the Use of an Electronic Medication Administration Record (Emar) in Clinical Simulation, International Journal of Nursing Education Scholarship. (2019) 16, no. 1, 10.1515/ijnes-2019-0014, 2-s2.0-85072541826. 31539361

[45] Oetker-Black S. L. and Davis T. , Psychometric Evaluation of the Mock Code Self-Efficacy Scale, Nursing Education Perspectives. (2019) 40, no. 1, 35–40, 10.1097/01.NEP.0000000000000341, 2-s2.0-85058882032. 29851700

[46] Keers R. N. , Williams S. D. , Cooke J. , and Ashcroft D. M. , Causes of Medication Administration Errors in Hospitals: a Systematic Review of Quantitative and Qualitative Evidence, Drug Safety. (2013) 36, no. 11, 1045–1067, 10.1007/s40264-013-0090-2, 2-s2.0-84887165647. 23975331 PMC3824584

[47] Wakefield B. , Measurement of the Frequency and Source of Interruptions Occurring During Bedside Nursing Handover in the Intensive Care Unit: an Observational Study, Australian Critical Care. (2017) 30, no. 2, 10.1016/j.aucc.2016.11.002, 2-s2.0-85015076364. 28292416

[48] Guo D. , Analysis of Risk Factors and Prediction Model for Nursing Interruption Events During the Surgical Counts, 2023, Shanxi Medical University.

[49] Qi W. , Wang Y. , Wang Y. et al., Prediction of Postpartum Depression in Women: Development and Validation of Multiple Machine Learning Models, Journal of Translational Medicine. (2025) 23, no. 1, 10.1186/s12967-025-06289-6. PMC11887113 40055720

