# Supplementary material for: Identifying Nurses at Risk of Nursing Interruptions During Medication Administration Using Machine Learning: A Multicenter Cross‐Sectional Study
Source: J Nurs Manag. 2026 Apr 20;2026:4433675. doi: 10.1155/jonm/4433675 (PMC13095847; doi:10.1155/jonm/4433675)
Supplement: Supplementary file 1 — Supporting Information Additional supporting information can be found online in the Supporting Information section. [file JONM-2026-4433675-s001.zip › Supplementary Figure S1.docx]

| 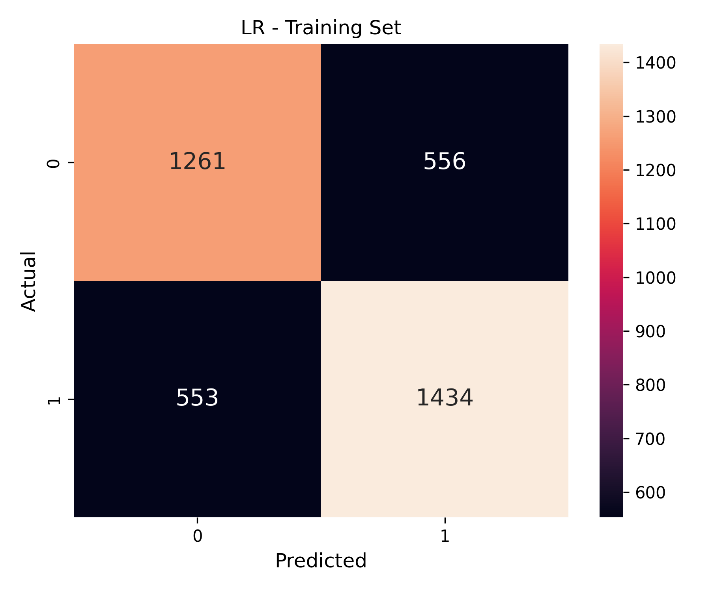  (A) LR model in the training set | (B) LR model in the internal test set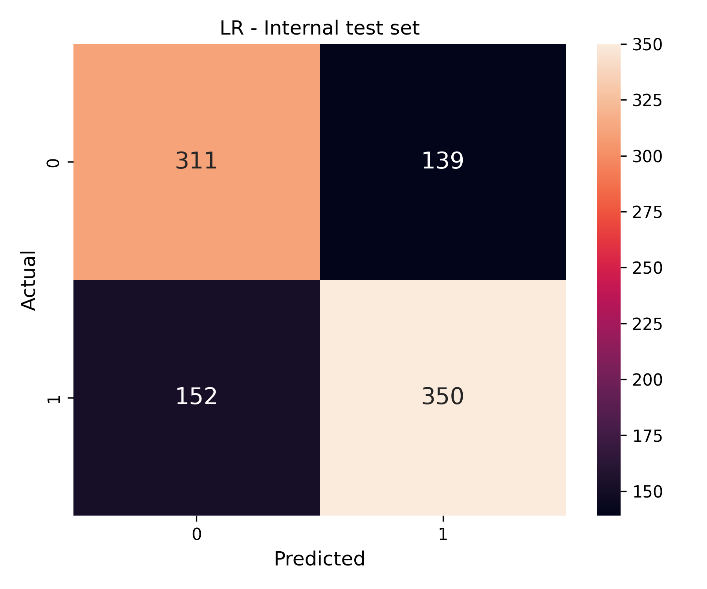 |
| --- | --- |
| 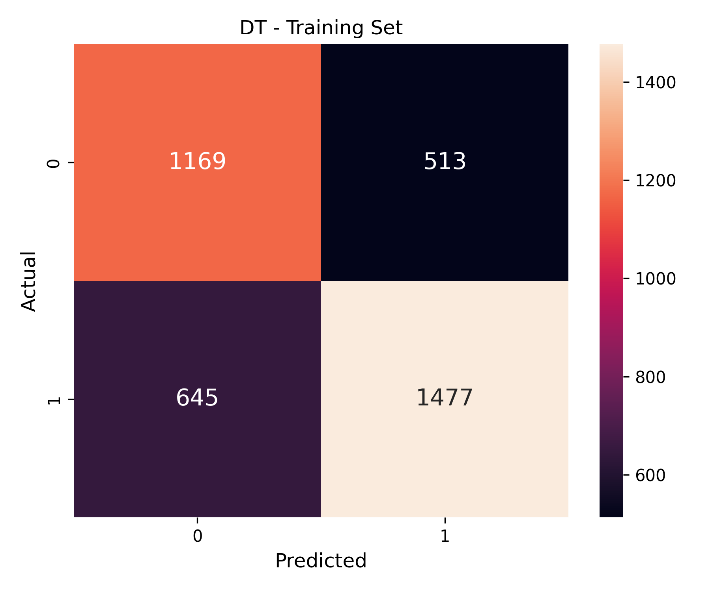  (C) DT model in the training set | 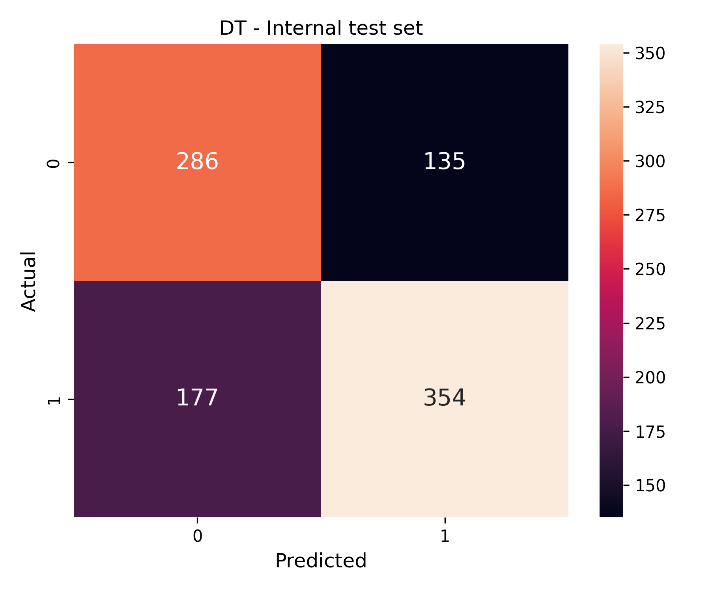  (D) DT model in the internal test set |
| (E) NB model in the training set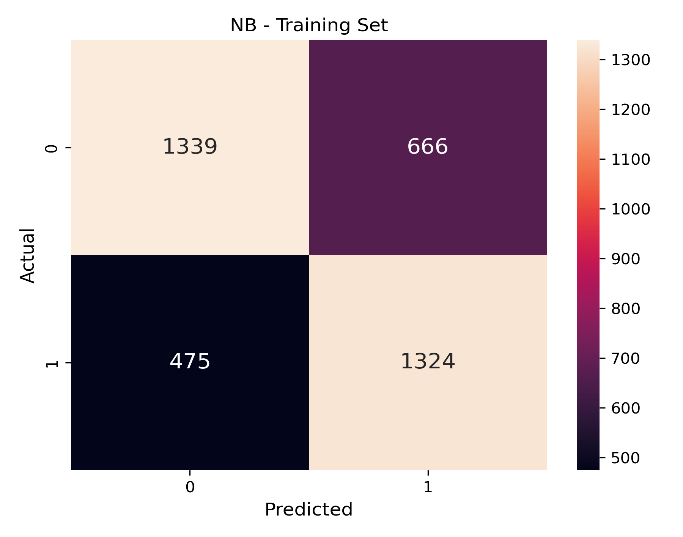 | (F) NB model in the internal test set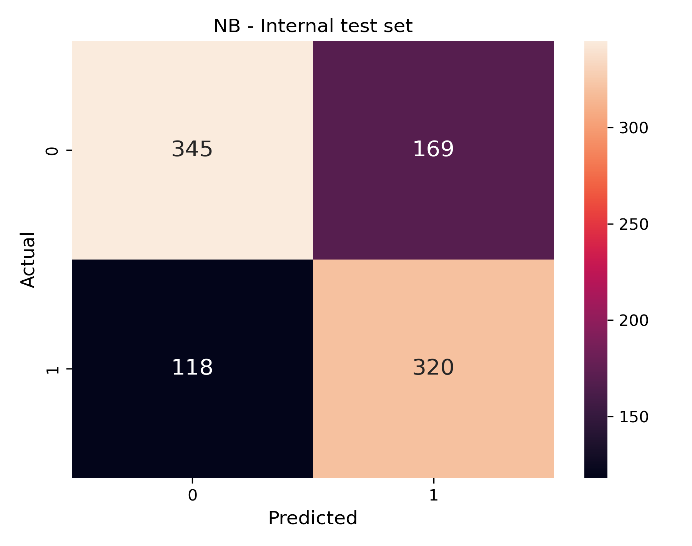 |

**Figure S1. Confusion matrices of the three machine learning models.** (A) LR model in the training set; (B) LR model in the internal test set; (C) DT model in the training set; (D) DT model in the internal test set; (E) NB model in the training set; (F) NB model in the internal test set.
Abbreviations: LR, Logistic Regression; DT, Decision Tree; NB, Naive Bayes.
