# Supplementary material for: Identifying Nurses at Risk of Nursing Interruptions During Medication Administration Using Machine Learning: A Multicenter Cross‐Sectional Study
Source: J Nurs Manag. 2026 Apr 20;2026:4433675. doi: 10.1155/jonm/4433675 (PMC13095847; doi:10.1155/jonm/4433675)
Supplement: Supplementary file 1 — Supporting Information Additional supporting information can be found online in the Supporting Information section. [file JONM-2026-4433675-s001.zip › Supplementary_Example 1.docx]

**Hypothetical Case:**
A single nurse works in the internal medicine department with a monthly income >10,000 yuan. Her resignation intention is "always," and she exercises >3 times/week. Her shift type is clinical quality oversight, shift time range is AM shift. Her behavior level of NI is 6, and general self-efficacy is 90. She experiences interruptions from doctors, colleagues, other hospital staff, patients, patients’ families, incorrect doctor’s orders，unreasonable zoning, and insufficient supplies, but not from the head nurse.

**Point Calculation:**

- Marital status (Single): 22.50 points
- Department (Internal Medicine): 48.75 points
- Monthly income (>10000): 46.25 points
- Resignation intention (Always): 100.00 points
- Physical exercise (>3 times/week): 27.50 points
- Shift Type (Clinical quality): 5.00 points
- Shift time (AM): 22.50 points
- Behavior level of NI (6): 90.00 points
- General self-efficacy (90): 85.00 points
- Interruptions (various sources): Doctors (42.50 points) + Head nurse (=0 point) + Colleagues (33.75 points) + Other staff (28.75 points) + Patients (61.25 points) + Patients’ family members (36.25 points) + Incorrect doctor’s orders (=15.00 points)+ Unreasonable functional zoning of departments (=15.00 points) + Insufficient medication supplies (16.25 points).

**Total Score:** 696.25 points
**Predicted Probability:** >95%
