# Supplementary material for: Identifying Nurses at Risk of Nursing Interruptions During Medication Administration Using Machine Learning: A Multicenter Cross‐Sectional Study
Source: J Nurs Manag. 2026 Apr 20;2026:4433675. doi: 10.1155/jonm/4433675 (PMC13095847; doi:10.1155/jonm/4433675)
Supplement: Supplementary file 1 — Supporting Information Additional supporting information can be found online in the Supporting Information section. [file JONM-2026-4433675-s001.zip › Supplementary_Figure_S2_Web_based_Calculators.docx]

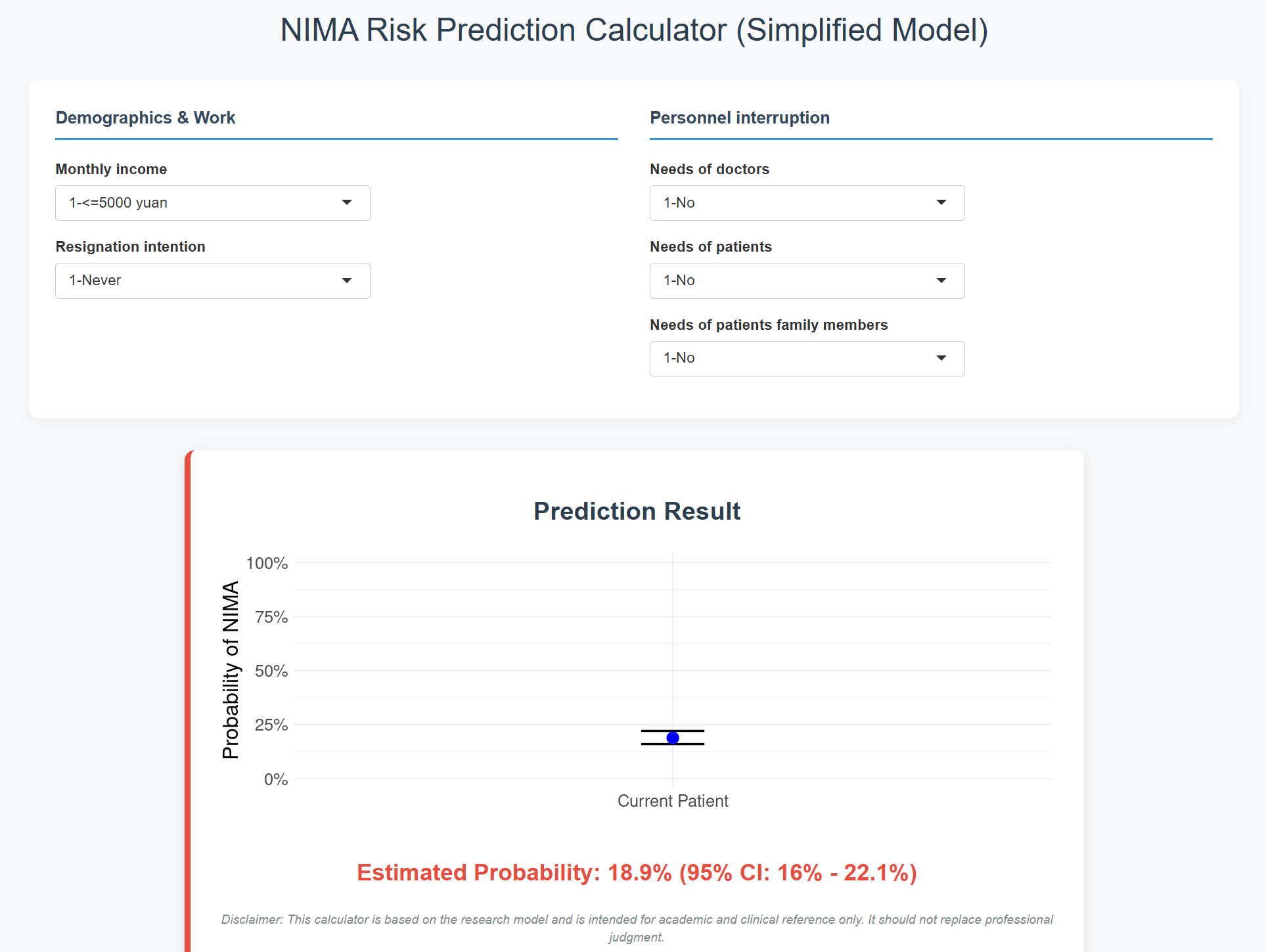


****Supplementary Figure S2-1.** User interface of the simplified 5-variable web-based NIMA risk prediction calculator.** The simplified model requires only 5 key predictors, providing a rapid and user-friendly tool for clinical assessment.


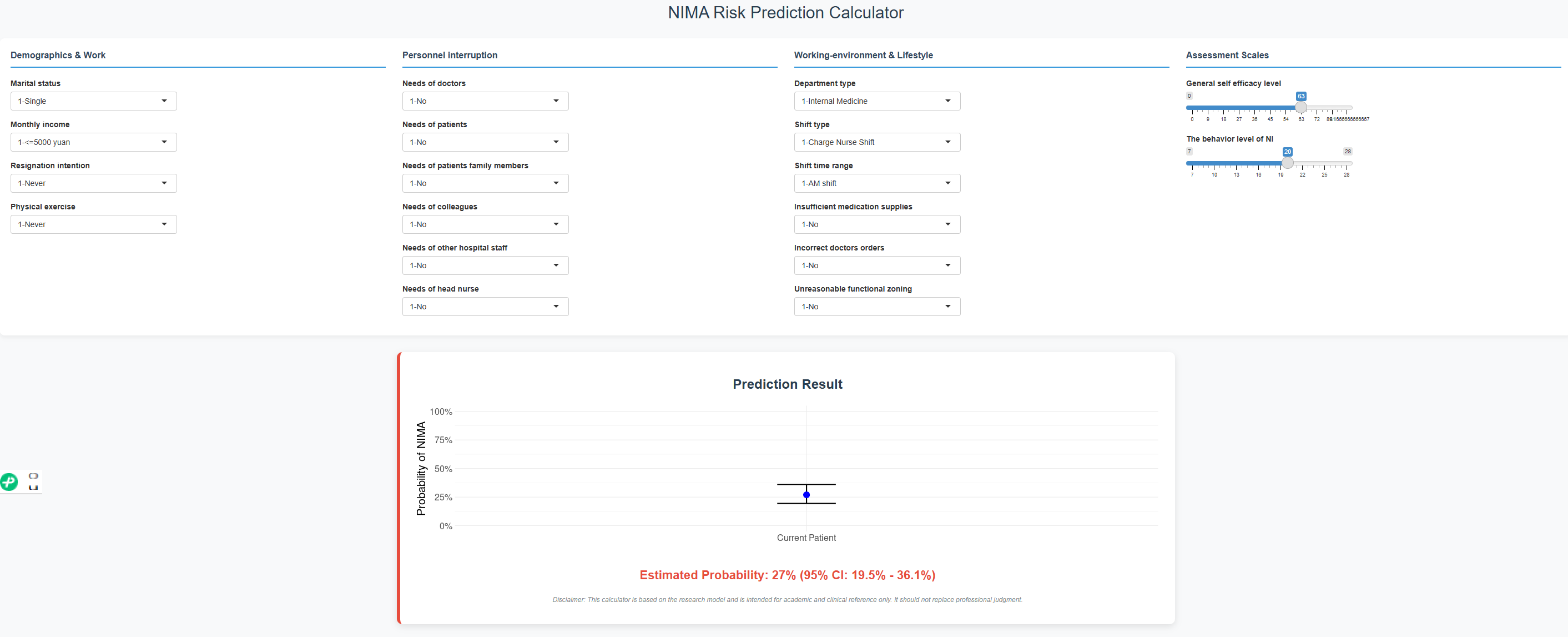


****Supplementary Figure S2-1.** User interface of the comprehensive 18-variable web-based NIMA risk prediction calculator. The full model includes all 18 predictors, designed for detailed and comprehensive risk evaluation.**
