# Supplementary material for: Identifying Nurses at Risk of Nursing Interruptions During Medication Administration Using Machine Learning: A Multicenter Cross‐Sectional Study
Source: J Nurs Manag. 2026 Apr 20;2026:4433675. doi: 10.1155/jonm/4433675 (PMC13095847; doi:10.1155/jonm/4433675)
Supplement: Supplementary file 1 — Supporting Information Additional supporting information can be found online in the Supporting Information section. [file JONM-2026-4433675-s001.zip › Supplementary_Table 3_Collinearity analysis.docx]

| **Model** | | **Non-standardized coefficient** | | **standardized coefficient** | **t** | **P** | **Statistics of collinearity** | |
| --- | --- | --- | --- | --- | --- | --- | --- | --- |
|  |  | **B** | **Standard error** | **β** |  |  | **Allowable error** | **Variance Inflation Factor** |
| 1 | （Constant） | -0.109 | 0.068 |  | -1.599 | 0.110 |  |  |
|  | X1 | -0.021 | 0.005 | -0.052 | -3.996 | 0.000 | 0.966 | **1.035** |
|  | X5 | -0.038 | 0.015 | -0.033 | -2.487 | 0.013 | 0.932 | **1.073** |
|  | X10 | 0.057 | 0.011 | 0.067 | 5.020 | 0.000 | 0.921 | **1.085** |
|  | X13 | 0.061 | 0.007 | 0.115 | 8.148 | 0.000 | 0.838 | **1.193** |
|  | X16 | 0.034 | 0.011 | 0.039 | 2.969 | 0.003 | 0.960 | **1.042** |
|  | X18 | 0.001 | 0.005 | 0.003 | 0.193 | 0.847 | 0.908 | **1.102** |
|  | X19 | -0.013 | 0.005 | -0.037 | -2.736 | 0.006 | 0.892 | **1.122** |
|  | X22 | -0.009 | 0.001 | -0.094 | -6.803 | 0.000 | 0.872 | **1.147** |
|  | X31 | 0.002 | 0.000 | 0.051 | 3.883 | 0.000 | 0.954 | **1.049** |
|  | X34 | 0.116 | 0.019 | 0.112 | 6.012 | 0.000 | 0.475 | **2.104** |
|  | X35 | -0.070 | 0.020 | -0.062 | -3.519 | 0.000 | 0.534 | **1.873** |
|  | X36 | 0.068 | 0.019 | 0.067 | 3.603 | 0.000 | 0.486 | **2.059** |
|  | X37 | 0.051 | 0.020 | 0.047 | 2.556 | 0.011 | 0.486 | **2.059** |
|  | X38 | 0.153 | 0.019 | 0.153 | 8.123 | 0.000 | 0.471 | **2.124** |
|  | X39 | 0.084 | 0.019 | 0.084 | 4.455 | 0.000 | 0.468 | **2.137** |
|  | X41 | -0.065 | 0.022 | -0.039 | -2.931 | 0.003 | 0.935 | **1.070** |
|  | X45 | 0.030 | 0.015 | 0.029 | 1.941 | 0.052 | 0.735 | **1.361** |
|  | X48 | 0.037 | 0.015 | 0.036 | 2.400 | 0.016 | 0.727 | **1.375** |
| Dependent variable：NIMA | | | | | | | | |

| **Collinearity diagnostics** | | | | | | | | | | | | | | | | | | | | | | |
| --- | --- | --- | --- | --- | --- | --- | --- | --- | --- | --- | --- | --- | --- | --- | --- | --- | --- | --- | --- | --- | --- | --- |
| **Model** | **Dimension** | **Eigenvalue** | **Condition**  **Index** | **variance proportion** | | | | | | | | | | | | | | | | | | |
|  |  |  |  | **（Constant）** | **X1** | **X5** | **X10** | **X13** | **X16** | **X18** | **X19** | **X22** | **X31** | **X34** | **X35** | **X36** | **X37** | **X38** | **X39** | **X41** | **X45** | **X48** |
| 1 | 1 | 17.142 | **1.000** | 0.00 | 0.00 | 0.00 | 0.00 | 0.00 | 0.00 | 0.00 | 0.00 | 0.00 | 0.00 | 0.00 | 0.00 | 0.00 | 0.00 | 0.00 | 0.00 | 0.00 | 0.00 | 0.00 |
|  | 2 | 0.365 | **6.856** | 0.00 | 0.13 | 0.00 | 0.00 | 0.01 | 0.01 | 0.08 | 0.10 | 0.00 | 0.00 | 0.01 | 0.01 | 0.01 | 0.01 | 0.01 | 0.01 | 0.00 | 0.00 | 0.00 |
|  | 3 | 0.231 | **8.613** | 0.00 | 0.39 | 0.00 | 0.00 | 0.00 | 0.01 | 0.15 | 0.32 | 0.01 | 0.01 | 0.00 | 0.00 | 0.00 | 0.00 | 0.00 | 0.00 | 0.00 | 0.00 | 0.00 |
|  | 4 | 0.207 | **9.106** | 0.00 | 0.43 | 0.01 | 0.02 | 0.01 | 0.10 | 0.00 | 0.04 | 0.04 | 0.03 | 0.01 | 0.00 | 0.01 | 0.00 | 0.00 | 0.00 | 0.00 | 0.00 | 0.00 |
|  | 5 | 0.165 | **10.190** | 0.00 | 0.00 | 0.00 | 0.00 | 0.08 | 0.04 | 0.55 | 0.41 | 0.00 | 0.00 | 0.00 | 0.00 | 0.00 | 0.00 | 0.00 | 0.00 | 0.00 | 0.00 | 0.00 |
|  | 6 | 0.140 | **11.063** | 0.00 | 0.00 | 0.01 | 0.00 | 0.34 | 0.11 | 0.16 | 0.09 | 0.00 | 0.01 | 0.01 | 0.01 | 0.01 | 0.02 | 0.01 | 0.01 | 0.00 | 0.02 | 0.02 |
|  | 7 | 0.114 | **12.261** | 0.00 | 0.00 | 0.01 | 0.04 | 0.09 | 0.62 | 0.00 | 0.00 | 0.05 | 0.07 | 0.00 | 0.00 | 0.00 | 0.00 | 0.00 | 0.00 | 0.00 | 0.04 | 0.02 |
|  | 8 | 0.099 | **13.169** | 0.00 | 0.00 | 0.01 | 0.35 | 0.10 | 0.02 | 0.00 | 0.01 | 0.06 | 0.03 | 0.01 | 0.01 | 0.00 | 0.00 | 0.00 | 0.00 | 0.00 | 0.15 | 0.08 |
|  | 9 | 0.091 | **13.715** | 0.00 | 0.00 | 0.01 | 0.30 | 0.20 | 0.02 | 0.00 | 0.00 | 0.06 | 0.01 | 0.00 | 0.01 | 0.00 | 0.00 | 0.02 | 0.01 | 0.00 | 0.21 | 0.09 |
|  | 10 | 0.071 | **15.519** | 0.00 | 0.01 | 0.00 | 0.05 | 0.01 | 0.00 | 0.01 | 0.00 | 0.00 | 0.00 | 0.05 | 0.21 | 0.01 | 0.02 | 0.17 | 0.20 | 0.01 | 0.00 | 0.01 |
|  | 11 | 0.062 | **16.572** | 0.00 | 0.00 | 0.01 | 0.02 | 0.00 | 0.01 | 0.01 | 0.00 | 0.24 | 0.60 | 0.00 | 0.01 | 0.00 | 0.00 | 0.00 | 0.00 | 0.01 | 0.10 | 0.09 |
|  | 12 | 0.056 | **17.438** | 0.00 | 0.00 | 0.70 | 0.14 | 0.02 | 0.00 | 0.00 | 0.01 | 0.05 | 0.04 | 0.00 | 0.02 | 0.03 | 0.00 | 0.00 | 0.01 | 0.00 | 0.05 | 0.02 |
|  | 13 | 0.056 | **17.522** | 0.00 | 0.00 | 0.05 | 0.00 | 0.01 | 0.00 | 0.00 | 0.00 | 0.06 | 0.15 | 0.01 | 0.01 | 0.00 | 0.03 | 0.00 | 0.01 | 0.00 | 0.38 | 0.55 |
|  | 14 | 0.046 | **19.217** | 0.00 | 0.00 | 0.02 | 0.02 | 0.02 | 0.00 | 0.00 | 0.00 | 0.00 | 0.00 | 0.13 | 0.25 | 0.27 | 0.37 | 0.03 | 0.03 | 0.00 | 0.02 | 0.03 |
|  | 15 | 0.044 | **19.836** | 0.00 | 0.01 | 0.03 | 0.01 | 0.00 | 0.00 | 0.00 | 0.00 | 0.00 | 0.01 | 0.01 | 0.01 | 0.60 | 0.49 | 0.02 | 0.03 | 0.00 | 0.00 | 0.01 |
|  | 16 | 0.042 | **20.160** | 0.00 | 0.00 | 0.01 | 0.00 | 0.00 | 0.00 | 0.00 | 0.00 | 0.00 | 0.01 | 0.75 | 0.45 | 0.00 | 0.01 | 0.00 | 0.05 | 0.00 | 0.00 | 0.00 |
|  | 17 | 0.033 | **22.767** | 0.00 | 0.00 | 0.00 | 0.01 | 0.00 | 0.00 | 0.00 | 0.00 | 0.02 | 0.00 | 0.01 | 0.01 | 0.05 | 0.05 | 0.63 | 0.63 | 0.02 | 0.00 | 0.00 |
|  | 18 | 0.027 | **25.098** | 0.02 | 0.01 | 0.05 | 0.00 | 0.03 | 0.01 | 0.01 | 0.00 | 0.25 | 0.01 | 0.00 | 0.00 | 0.00 | 0.00 | 0.10 | 0.01 | 0.59 | 0.00 | 0.09 |
|  | 19 | 0.008 | **47.588** | 0.98 | 0.02 | 0.08 | 0.02 | 0.09 | 0.04 | 0.02 | 0.00 | 0.15 | 0.03 | 0.00 | 0.00 | 0.00 | 0.00 | 0.00 | 0.00 | 0.36 | 0.00 | 0.00 |
| Dependent variable：NIMA | | | | | | | | | | | | | | | | | | | | | | |
