# Supplementary material for: Identifying Nurses at Risk of Nursing Interruptions During Medication Administration Using Machine Learning: A Multicenter Cross‐Sectional Study
Source: J Nurs Manag. 2026 Apr 20;2026:4433675. doi: 10.1155/jonm/4433675 (PMC13095847; doi:10.1155/jonm/4433675)
Supplement: Supplementary file 1 — Supporting Information Additional supporting information can be found online in the Supporting Information section. [file JONM-2026-4433675-s001.zip › Supplementary_Table_1_Univariate_Analysis.docx]

| **Table 1 The univariate analyses of the potential predictive factors associated with NIMA（n=4758）** | | | | | | |
| --- | --- | --- | --- | --- | --- | --- |
| **Variables** | **Items** | **n（%）** | **n（%）/X±S** | | **X^2^/t** | ***P*** |
|  |  |  | **No NIMA**  **2278（47.9%）** | **NIMA**  **2480（52.1%）** |  |  |
| **1. Sociodemographic information** | | | | | | |
| X1 Department type | Internal Medicine | 180（38.0%） | 850（37.3%） | 959（38.7%） | 51.557 | ＜0.001 |
|  | General Surgery | 142（30.0%） | 630（27.7%） | 796（32.1%） |  |  |
|  | Obstetrics & Pediatrics | 648（13.6%） | 311（13.7%） | 337（13.6%） |  |  |
|  | Emergency & Critical Care | 521（10.9%） | 256（11.2%） | 265（10.7%） |  |  |
|  | Others | 354（7.4%） | 231（10.1%） | 123（5%） |  |  |
| X2 Gender | Male | 148（3.1%） | 79（3.5%） | 69（2.8%） | 1.852 | 0.174 |
|  | Female | 4610（96.9%） | 2199（96.5%） | 2411（97.2%） |  |  |
| X3 Age |  | 32.68±6.93 | 32.56±7.23 | 32.79±6.63 | -1.166 | 0.244 |
| X4 Educational level | Associate's Degree or Below | 869（18.3%） | 483（21.2%） | 386（15.6%） | 44.961 | ＜0.001 |
|  | Bachelor's Degree | 373（78.6%） | 175（76.9%） | 1986（80.1%） |  |  |
|  | Master's Degree or Above | 151（3.2%） | 43（1.9%） | 108（4.4%） |  |  |
| X5 Marital status | Single | 126（26.5%） | 619（27.2%） | 643（25.9%） | 0.945 | 0.331 |
|  | Currently Married | 349（73.5%） | 165（72.8%） | 1837（74.1%） |  |  |
| X6 Number of children | 0 | 156（33.0%） | 777（34.1%） | 791（31.9%） | 2.642 | 0.267 |
|  | 1 | 170（35.9%） | 804（35.3%） | 902（36.4%） |  |  |
|  | ≥2 | 148（31.2%） | 697（30.6%） | 787（31.7%） |  |  |
| X7 Professional title | Nurse | 662（13.9%） | 372（16.3%） | 290（11.7%） | 24.980 | ＜0.001 |
|  | Nurse practitioner | 156（32.9%） | 758（33.3%） | 807（32.5%） |  |  |
|  | Supervisor nurse | 253（53.2%） | 114（50.4%） | 1383（55.8%） |  |  |
| X8 Position | Head nurse | 364（7.7%） | 149（6.5%） | 215（8.7%） | 17.625 | 0.001 |
|  | Quality control team leader | 353（7.4%） | 160（7%） | 193（7.8%） |  |  |
|  | Head teacher | 183（3.8%） | 72（3.2%） | 111（4.5%） |  |  |
|  | Other | 114（2.4%） | 1834（80.5%） | 1910（77%） |  |  |
|  | No | 374（78.7%） | 63（2.8%） | 51（2.1%） |  |  |
| X9 Service years | ≤5 year | 117（24.7%） | 596（26.2%） | 579（23.3%） | 6.317 | 0.042 |
|  | 6-10 year | 125（26.5%） | 607（26.6%） | 652（26.3%） |  |  |
|  | 11-15 year | 232（48.8%） | 107（47.2%） | 1249（50.4%） |  |  |
| X10 Monthly income | ≤5000 yuan | 149（31.4%） | 826（36.3%） | 667（26.9%） | 51.087 | ＜0.001 |
|  | 5000-10000 yuan | 283（59.5%） | 1275（56%） | 1556（62.7%） |  |  |
|  | ＞10000yuan | 434（9.1%） | 177（7.8%） | 257（10.4%） |  |  |
| **2. Daily work and life information** | | | | | | |
| X11 The understanding level of NIMA | Not at all | 92（1.9%） | 44（1.9%） | 48（1.9%） | 98.227 | ＜0.001 |
|  | Slightly | 846（17.8%） | 347（15.2%） | 499（20.1%） |  |  |
|  | Normal | 1810（38.0%） | 784（34.4%） | 1026（41.4%） |  |  |
|  | Better | 1538（32.3%） | 794（34.9%） | 744（30%） |  |  |
|  | Very | 472（9.9%） | 309（13.6%） | 163（6.6%） |  |  |
| X13 Resignation intention | Never | 1057（22.2%） | 678（29.8%） | 379（15.3%） | 250.189 | ＜0.001 |
|  | Rarely | 1221（25.7%） | 664（29.1%） | 557（22.5%） |  |  |
|  | Sometimes | 2114（44.4%） | 831（36.5%） | 1283（51.7%） |  |  |
|  | Often | 300（6.3%） | 82（3.6%） | 218（8.8%） |  |  |
|  | Always | 66（1.4%） | 23（1.0%） | 43（1.7%） |  |  |
| X12 Actual working hours | ＜36h/w | 148（3.1%） | 87（3.8%） | 61（2.5%） | 24.923 | ＜0.001 |
|  | 36-40h/w | 2062（43.3%） | 1042（45.7%） | 1020（41.1%） |  |  |
|  | 41-48h/w | 1784（37.5%） | 781（34.3%） | 1003（40.4%） |  |  |
|  | ＞48h/w | 764（16.1%） | 368（16.2%） | 396（16%） |  |  |
| X14 Sleep duration | ≤7h/d | 3456（72.6%） | 1598（70.1%） | 1858（74.9%） | 13.593 | ＜0.001 |
|  | ＞7h/d | 1302（27.4%） | 680（29.9%） | 622（25.1%） |  |  |
| X15 Seep problems | No | 2040（42.9%） | 1091（47.9%） | 949（38.3%） | 44.927 | ＜0.001 |
|  | Yes | 2718（57.1%） | 1187（52.1%） | 1531（61.7%） |  |  |
| X16Physical exercise frequency | Never | 2841（59.7%） | 1367（60%） | 1474（59.4%） | 0.316 | ＜0.001 |
|  | 1-2 times/week | 1703（35.8%） | 812（35.6%） | 891（35.9%） |  |  |
|  | ＞3 times/week | 214（4.5%） | 99（4.3%） | 115（4.6%） |  |  |
| X17Physical exercise duration | ＜30 min | 3826（80.4%） | 1832（80.4%） | 1994（80.4%） | 0.127 | 0.938 |
|  | 30-60 min | 826（17.4%） | 397（17.4%） | 429（17.3%） |  |  |
|  | ＞60 min | 106（2.2%） | 49（2.2%） | 57（2.3%） |  |  |
| **3. Previous Shift Variables** | | | | | | |
| X18 Shift Type | Charge Nurse Shift | 579（12.2%） | 300（13.2%） | 279（11.3%） | 20.445 | ＜0.001 |
|  | Primary Nursing Duty | 2543（53.4%） | 1157（50.8%） | 1386（55.9%） |  |  |
|  | Support Nurse Shift | 274（5.8%） | 151（6.6%） | 123（5%） |  |  |
|  | Clinical Quality Oversight | 179（3.8%） | 75（3.3%） | 104（4.2%） |  |  |
|  | Other Specialty Roles | 1183（24.9%） | 595（26.1%） | 588（23.7%） |  |  |
| X19  Shift time range | AM shift | 1814（38.1%） | 840（36.9%） | 974（39.3%） | 16.764 | 0.002 |
|  | PM shift | 453（9.5%） | 208（9.1%） | 245（9.9%） |  |  |
|  | Night shift | 693（14.6%） | 370（16.2%） | 323（13%） |  |  |
|  | Day Duty | 1371（28.8%） | 634（27.8%） | 737（29.7%） |  |  |
|  | Others | 427（9.0%） | 226（9.9%） | 201（8.1%） |  |  |
| **4. Risk Factor Variables for NIMA** | | | | | | |
| X20 The knowledge level of NI | | 31.63±9.90 | 32.63±10.45 | 30.71±9.29 | 6.672 | ＜0.001 |
| X21 The attitude level of NI | | 37.44±7.30 | 38.13±7.32 | 36.82±7.25 | 6.218 | ＜0.001 |
| X22 The behavior level of NI | | 19.64±5.39 | 20.74±5.54 | 18.62±5.04 | 13.764 | ＜0.001 |
| X23Unfamiliarity with commonly used department drugs | No | 514（10.8%） | 269（11.8%） | 245（9.9%） | 4.588 | 0.032 |
|  | Yes | 4244（89.2%） | 2009（88.2%） | 2235（90.1%） |  |  |
| X24Unfamiliarity with commonly used related equipment | No | 455（9.6%） | 255（11.2%） | 200（8.1%） | 13.446 | ＜0.001 |
|  | Yes | 4303（90.4%） | 2023（88.8%） | 2280（91.9%） |  |  |
| X25 Handling personal matters | No | 3575（75.1%） | 1847（81.1%） | 1728（69.7%） | 82.636 | ＜0.001 |
|  | Yes | 1183（24.9%） | 431（18.9%） | 752（30.3%） |  |  |
| X26 Work-related confusion | No | 1509（31.7%） | 705（30.9%） | 804（32.4%） | 1.187 | 0.276 |
|  | Yes | 3249（68.3%） | 1573（69.1%） | 1676（67.6%） |  |  |
| X27 Circadian rhythm disturbances | No | 1771（37.2%） | 1029（45.2%） | 742（29.9%） | 118.201 | ＜0.001 |
|  | Yes | 2987（62.8%） | 1249（54.8%） | 1738（70.1%） |  |  |
| X28 Physical discomfort | No | 2173（45.7%） | 1258（55.2%） | 915（36.9%） | 160.758 | ＜0.001 |
|  | Yes | 2585（54.3%） | 1020（44.8%） | 1565（63.1%） |  |  |
| X29 Lack of concentration | No | 3593（75.5%） | 1899（83.4%） | 1694（68.3%） | 145.571 | ＜0.001 |
|  | Yes | 1165（24.5%） | 379（16.6%） | 786（31.7%） |  |  |
| X30 Risk perception of nursing environment | | 87.57±23.31 | 83.65±24.92 | 91.17±21.11 | -11.173 | 0.014 |
| X31 General self-efficacy | | 58.96±16.95 | 58.04±16.65 | 59.81±16.23 | -3.580 | ＜0.001 |
| X32 Mental workload | | 29.31±4.86 | 29.94±5.03 | 28.73±4.63 | 8.629 | ＜0.001 |
| X33 Job burnout |  | 82.24±24.07 | 78.22±24.31 | 83.92±23.24 | -11.164 | ＜0.001 |
| X34 Needs of doctors | No | 2999（63.0%） | 1812（79.5%） | 1187  （47.9%） | 511.415 | ＜0.001 |
|  | Yes | 1759（37.0%） | 466（20.5%） | 1293（52.1%） |  |  |
| X35 Needs of head nurse | No | 3468（72.9%） | 1903（83.5%） | 1565（63.1%） | 250.865 | ＜0.001 |
|  | Yes | 1290（27.1%） | 375（16.5%） | 915（36.9%） |  |  |
| X36 Needs of  colleagues | No | 2799（58.8%） | 1703（74.8%） | 1096（44.2%） | 457.975 | ＜0.001 |
|  | Yes | 1959（41.2%） | 575（25.2%） | 1384（55.8%） |  |  |
| X37 Needs of other hospital staffs | No | 3314（69.7%） | 1910（83.8%） | 1404（56.6%） | 416.570 | ＜0.001 |
|  | Yes | 1444（30.3%） | 368（16.2%） | 1076（43.4%） |  |  |
| X38 Needs of patients | No | 2356（49.5%） | 1562（68.6%） | 794（32%） | 634.633 | ＜0.001 |
|  | Yes | 2402（50.5%） | 716（31.4%） | 1686（68%） |  |  |
| X39 Needs of patients families | No | 2659（55.9%） | 1674（73.5%） | 985（39.7%） | 549.166 | ＜0.001 |
|  | Yes | 2099（44.1%） | 604（26.5%） | 1495（60.3%） |  |  |
| X40 Needs of others | No | 3680（77.3%） | 1992（87.4%） | 1688（68.1%） | 254.506 | ＜0.001 |
|  | Yes | 1078（22.7%） | 286（12.6%） | 792（31.9%） |  |  |
| X41 Incorrect doctor’s orders | No | 2107（44.3%） | 1250（54.9%） | 857（34.6%） | 198.629 | ＜0.001 |
|  | Yes | 2651（55.7%） | 1028（45.1%） | 1623（65.4%） |  |  |
| X42 Incomplete patient identification | No | 2754（57.9%） | 1507（66.2%） | 1247（50.3%） | 122.700 | ＜0.001 |
|  | Yes | 2004（42.1%） | 771（33.8%） | 1233（49.7%） |  |  |
| X43 Erroneous patient information | No | 2954（62.1%） | 1600（70.2%） | 1354（54.6%） | 123.388 | ＜0.001 |
|  | Yes | 1804（37.9%） | 678（29.8%） | 1126（45.4%） |  |  |
| X44 Good safety culture | No | 605（12.7%） | 301（13.2%） | 304（12.3%） | 0.976 | 0.323 |
|  | Yes | 4153（87.3%） | 1977（86.8%） | 2176（87.7%） |  |  |
| X45 Unreasonable functional zoning of departments | No | 2854（60.0%） | 1605（70.5%） | 1249（50.4%） | 199.722 | ＜0.001 |
|  | Yes | 1904（40.0%） | 673（29.5%） | 1231（49.6%） |  |  |
| X46 Noise pollution | No | 2393（50.3%） | 1408（61.8%） | 985（39.7%） | 231.783 | ＜0.001 |
|  | Yes | 2365（49.7%） | 870（38.2%） | 1495（60.3%） |  |  |
| X47 Uncomfortable lighting conditions | No | 2908（61.1%） | 1602（70.3%） | 1306（52.7%） | 155.891 | ＜0.001 |
|  | Yes | 1850（38.9%） | 676（29.7%） | 1174（47.3%） |  |  |
| X48 Insufficient medication supplies | No | 481（10.1%） | 244（10.7%） | 237（9.6%） | 1.742 | 0.187 |
|  | Yes | 4277（89.9%） | 2034（89.3%） | 2243（90.4%） |  |  |
| X49 Availability of office equipment | No | 1537（32.3%） | 939（41.2%） | 598（24.1%） | 158.905 | ＜0.001 |
|  | Yes | 3221（67.7%） | 1339（58.8%） | 1882（75.9%） |  |  |
| X50 Availability of drug-related instruments | No | 2307（48.5%） | 1310（57.5%） | 997（40.2%） | 142.358 | ＜0.001 |
|  | Yes | 2451（51.5%） | 968（42.5%） | 1483（59.8%） |  |  |
| X51 Availability of auxiliary facilities | No | 2102（44.2%） | 1224（53.7%） | 878（35.4%） | 161.735 | ＜0.001 |
|  | Yes | 2656（55.8%） | 1054（46.3%） | 1602（64.6%） |  |  |
| X52 Information system issues | No | 1394（29.3%） | 865（38%） | 529（21.3%） | 158.739 | ＜0.001 |
|  | Yes | 3364（70.7%） | 1413（62%） | 1951（78.7%） |  |  |
