# Supplementary material for: Identifying Nurses at Risk of Nursing Interruptions During Medication Administration Using Machine Learning: A Multicenter Cross‐Sectional Study
Source: J Nurs Manag. 2026 Apr 20;2026:4433675. doi: 10.1155/jonm/4433675 (PMC13095847; doi:10.1155/jonm/4433675)
Supplement: Supplementary file 1 — Supporting Information Additional supporting information can be found online in the Supporting Information section. [file JONM-2026-4433675-s001.zip › Supplementary_Table_2_Training_vs_Test_Set.docx]

| **Table 2 Comparison of the training set and the internal test set (n=4758)** | | | | | |
| --- | --- | --- | --- | --- | --- |
| **Variables** | **Items** | **n（%）/X±S** | | **X^2^/t** | ***P*** |
|  |  | **Training set**  **3806（80.0%）** | **Internal test set**  **952（20.0%）** |  |  |
| **1. Sociodemographic information** | | | | | |
| X1 Department type | Internal Medicine | 1440（37.8%） | 369（38.8%） | 1.751 | 0.782 |
|  | General Surgery | 1148（30.2%） | 278（29.2%） |  |  |
|  | Obstetrics & Pediatrics | 511（13.4%） | 137（14.4%） |  |  |
|  | Emergency & Critical Care | 417（11.0%） | 104（10.9%） |  |  |
|  | Others | 290（7.6%） | 64（6.7%） |  |  |
| X2Gender | Male | 125（3.3%） | 23（2.4%） | 1.905 | 0.168 |
|  | Female | 3681（97.6%） | 929（96.7%） |  |  |
| X3 Age |  | 32.77±6.96 | 32.32±6.80 | -1.771 | 0.077 |
| X4 Educational level | Associate's Degree or Below | 688（18.1%） | 181（19.0%） | 0.736 | 0.865 |
|  | Bachelor's Degree | 2997（78.7%） | 741（77.8%） |  |  |
|  | Master's Degree or Above | 121（3.2%） | 30（3.2%） |  |  |
| X5 Marital status | Single | 990（26.0%） | 272（28.6%） | 2.561 | 0.110 |
|  | Currently Married | 2816（74.0%） | 680（71.4%） |  |  |
| X6 Number of children | 0 | 1233（32.4%） | 335（35.2%） | 3.646 | 0.162 |
|  | 1 | 1365（35.9%） | 341（35.8%） |  |  |
|  | ≥2 | 1208（31.7%） | 276（29.0%） |  |  |
| X7 Professional title | Nurse | 519（13.6%） | 143（15.0%） | 2.383 | 0.497 |
|  | Nurse practitioner | 1249（32.8%） | 316（33.2%） |  |  |
|  | Supervisor nurse | 1845（48.5%） | 453（47.6%） |  |  |
| X8 Position | Head nurse | 193（5.1%） | 40（4.2%） |  |  |
|  | Quality control team leader | 287（7.5%） | 77（8.1%） | 1.391 | 0.846 |
|  | Head teacher | 279（7.3%） | 74（7.8%） |  |  |
|  | other | 148（3.9%） | 35（3.7%） |  |  |
|  | No | 3092（81.2%） | 766（80.5%） |  |  |
| X9 Service years | ≤5 year | 932（24.5%） | 243（25.5%） | 1.186 | 0.553 |
|  | 6-10 year | 1000（26.3%） | 259（27.2%） |  |  |
|  | 11-15 year | 1874（49.2%） | 450（47.3%） |  |  |
| X10 Monthly income | ≤5000 yuan | 1183（31.1%） | 310（32.6%） | 0.782 | 0.676 |
|  | 5000-10000 yuan | 2275（59.8%） | 556（58.4%） |  |  |
|  | ＞10000yuan | 348（9.1%） | 86（9.0%） |  |  |
| **2. Daily work and life information** | | | | | |
| X11 The understanding level of NIMA | Not at all | 70（1.8%） | 22（2.3%） |  |  |
|  | Slightly | 658（17.3%） | 165（19.7%） | 6.594 | 0.159 |
|  | Normal | 1475（38.8%） | 419（35.2%） |  |  |
|  | Better | 1232（32.4%） | 266（32.1%） |  |  |
|  | Very | 371（9.7%） | 80（10.6%） |  |  |
| X13 Resignation intention | Never | 841（22.1%） | 216（22.7%） | 5.378 | 0.251 |
|  | Rarely | 954（25.1%） | 267（28.0%） |  |  |
|  | Sometimes | 714（45.0%） | 400（42.0%） |  |  |
|  | Often | 246（6.5%） | 54（5.7%） |  |  |
|  | Always | 51（1.3%） | 15（1.6%） |  |  |
| X12 Actual working hours | ＜36h/w | 118（3.1%） | 30（3.2%） | 1.040 | 0.792 |
|  | 36-40h/w | 1636（43.0%） | 426（44.7%） |  |  |
|  | 41-48h/w | 1438（37.8%） | 346（36.3%） |  |  |
|  | ＞48h/w | 614（16.1%） | 150（15.8%） |  |  |
| X14 Sleep duration | ≤7h/d | 2762（72.6%） | 694（72.9%） | 0.042 | 0.838 |
|  | ＞7h/d | 1044（27.4%） | 258（27.1%） |  |  |
| X15 Seep problems | No | 1639（43.9%） | 401（42.1%） | 0.276 | 0.600 |
|  | Yes | 2167（56.9%） | 551（57.9%） |  |  |
| X16 Physical exercise frequency | Never | 2276（59.8%） | 565（59.3%） | 0.085 | 0.958 |
|  | 1-2 time/week | 1360（35.7%） | 343（36.0%） |  |  |
|  | ＞3 time/week | 170（4.5%） | 44（4.6%） |  |  |
| X17 Physical exercise duration | ＜30 min | 3062（80.5%） | 764（80.3%） | 1.946 | 0.378 |
|  | 30-60 min | 654（17.2%） | 172（18.1%） |  |  |
|  | ＞60 min | 90（2.4%） | 16（1.7%） |  |  |
| **3. Previous Shift Variables** | | | | | |
| X18 Shift Type | Charge Nurse Shift | 457（12.0%） | 122（12.8%） | 1.876 | 0.759 |
|  | Primary Nursing Duty | 2033（53.4%） | 510（53.6%） |  |  |
|  | Support Nurse Shift | 214（5.6%） | 60（6.3%） |  |  |
|  | Clinical Quality Oversight | 143（3.8%） | 36（3.8%） |  |  |
|  | Other Specialty Roles | 959（25.2%） | 224（23.5%） |  |  |
| X19 Shift time range | AM shift | 1464（38.5%） | 350（36.8%） | 5.460 | 0.243 |
|  | PM shift | 374（9.8%） | 79（8.3%） |  |  |
|  | Night shift | 541（14.2%） | 152（16.0%） |  |  |
|  | Day Duty | 1081（28.4%） | 290（30.5%） |  |  |
|  | Others | 346（9.1%） | 81（8.5%） |  |  |
| **4. Risk Factor Variables for NIMA** | | | | | |
| X20 The knowledge level of NI | 31.63±9.90 | 31.52±9.81 | 32.07±10.26 | 1.486 | 0.137 |
| X21 The attitude level of NI | 37.44±7.30 | 37.39±7.34 | 37.67±7.17 | 1.055 | 0.291 |
| X22 The behavior level of NI | 19.64±5.39 | 19.58±5.37 | 19.87±5.47 | 1.510 | 0.131 |
| X23 Unfamiliarity with commonly used department drugs | No | 398（10.5%） | 116（12.2%） | 2.359 | 0.125 |
|  | Yes | 3408（89.5%） | 836（87.8%） |  |  |
| X24 Unfamiliarity with commonly used related equipment | No | 356（9.4%） | 99（10.4%） | 0.963 | 0.327 |
|  | Yes | 3450（90.6%） | 853（89.6%） |  |  |
| X25 Handling personal matters | No | 2872（75.5%） | 703（73.8%） | 1.064 | 0.302 |
|  | Yes | 934（24.5%） | 249（26.2%） |  |  |
| X26 Work-related confusion | No | 1185（31.1%） | 324（34.0%） | 2.954 | 0.086 |
|  | Yes | 2621（68.9%） | 628（66.0%） |  |  |
| X27 Circadian rhythm disturbances | No | 1415（37.2%） | 356（37.4%） | 0.015 | 0.901 |
|  | Yes | 2391（62.8%） | 596（62.6%） |  |  |
| X28 Physical discomfort | No | 1734（45.6%） | 439（46.1%） | 0.094 | 0.759 |
|  | Yes | 2072（54.4%） | 513（53.9%） |  |  |
| X29 Lack of concentration | No | 2878（75.6%） | 715（75.1%） | 0.108 | 0.742 |
|  | Yes | 928（24.4%） | 237（24.9%） |  |  |
| X30 Risk perception of nursing environment |  | 87.65±23.12 | 87.26±24.13 | -2.464 | 0.642 |
| X31 General self-efficacy |  | 58.95±16.89 | 58.99±11.16 | 0.053 | 0.958 |
| X32 Mental workload |  | 29.27±4.82 | 29.43±5.03 | 0.879 | 0.380 |
| X33 Job burnout |  | 82.40±23.98 | 81.57±24.42 | -0.959 | 0.338 |
| X34 Needs of doctors | No | 2411（63.3%） | 588（61.8%） | 0.819 | 0.366 |
|  | Yes | 1395（36.7%） | 364（38.2%） |  |  |
| X35 Needs of head nurse | No | 2777（73.0%） | 691（72.6%） | 0.056 | 0.814 |
|  | Yes | 1029（27.0%） | 261（27.4%） |  |  |
| X36 Needs of  colleagues | No | 2232（58.6%） | 567（59.6%） | 0.263 | 0.608 |
|  | Yes | 1574（41.4%） | 385（40.4%） |  |  |
| X37 Needs of other hospital staffs | No | 2663（70.0%） | 651（68.4%） | 0.906 | 0.341 |
|  | Yes | 1143（30.0%） | 301（31.6%） |  |  |
| X38 Needs of patients | No | 1895（49.8%） | 428（48.4%） | 0.568 | 0.451 |
|  | Yes | 1911（50.2%） | 49.1（51.6%） |  |  |
| X39 Needs of patients families | No | 2133（56.1%） | 524（55.0%） | 0.328 | 0.567 |
|  | Yes | 1671（43.9%） | 428（45.0%） |  |  |
| X40 Needs of others | No | 2943（77.3%） | 737（77.4%） | 0.004 | 0.952 |
|  | Yes | 863（22.7%） | 215（22.6%） |  |  |
| X41 Incorrect doctor’s orders | No | 374（9.8%） | 107（11.2%） | 1.673 | 0.196 |
|  | Yes | 3432（90.2%） | 845（88.8%） |  |  |
| X42 Incomplete patient identification | No | 2202（57.9%） | 552（58.0%） | 0.005 | 0.943 |
|  | Yes | 1604（42.1%） | 400（42.0%） |  |  |
| X43 Erroneous patient information | No | 2346（61.6%） | 608（63.9%） | 1.603 | 0.205 |
|  | Yes | 1460（38.4%） | 344（36.1%） |  |  |
| X44 Good safety culture | No | 470（12.3%） | 135（14.2%） | 2.302 | 0.129 |
|  | Yes | 3336（87.7%） | 817（858%） |  |  |
| X45 Unreasonable functional zoning of departments | No | 2258（59.3%） | 596（62.6%） | 3.408 | 0.065 |
|  | Yes | 1548（40.7%） | 356（37.4%） |  |  |
| X46 Noise pollution | No | 1898（49.9%） | 495（52.0%） | 1.378 | 0.240 |
|  | Yes | 1908（50.1%） | 457（48.0%） |  |  |
| X47 Uncomfortable lighting conditions | No | 2311（60.7%） | 597（62.7%） | 1.269 | 0.260 |
|  | Yes | 1495（39.3%） | 355（37.3%） |  |  |
| X48 Insufficient medication supplies | No | 1688（44.4%） | 419（44.0%） | 0.035 | 0.851 |
|  | Yes | 2118（55.6%） | 533（56.0%） |  |  |
| X49 Availability of office equipment | No | 1211（31.8%） | 326（34.2%） | 2.049 | 0.152 |
|  | Yes | 2595（68.2%） | 626（65.8%） |  |  |
| X50 Availability of drug-related instruments | No | 1840（48.3%） | 467（49.1%） | 0.154 | 0.695 |
|  | Yes | 1966（51.7%） | 485（50.9%） |  |  |
| X51 Availability of auxiliary facilities | No | 1669（43.9%） | 433（45.5%） | 0.822 | 0.365 |
|  | Yes | 2137（56.1%） | 519（54.5%） |  |  |
| X52 Insufficient medication supplies | No | 1116（29.3%） | 278（29.2%） | 0.005 | 0.942 |
|  | Yes | 2690（70.7%） | 674（70.8%） |  |  |
| Y-NIMA Occurrence | No | 1815（47.7%） | 463（48.6%） | 0.273 | 0.601 |
|  | Yes | 1991（52.3%） | 489（51.4%） |  |  |
