# Supplementary material for: Identifying Nurses at Risk of Nursing Interruptions During Medication Administration Using Machine Learning: A Multicenter Cross‐Sectional Study
Source: J Nurs Manag. 2026 Apr 20;2026:4433675. doi: 10.1155/jonm/4433675 (PMC13095847; doi:10.1155/jonm/4433675)
Supplement: Supplementary file 1 — Supporting Information Additional supporting information can be found online in the Supporting Information section. [file JONM-2026-4433675-s001.zip › Supplementary_Table_4_Performance_of_Simplified_Model.docx]

**Table 4 Performance metrics of NIMA risk prediction models in the training and internal test set (Five predictor variables)**

| **Model** | **AUC**  **（95%CI）** | **Accuracy** | **Precision** | **Sensitivity** | **Specificity** |
| --- | --- | --- | --- | --- | --- |
| **Training set** | | | | | |
| LR model | 0.749  (0.749,0.751)** | 0.698 | 0.728 | 0.674 | 0.724 |
| **Internal test set** | | | | | |
| LR model | 0.752  (0.758, 0.760)** | 0.670 | 0.718 | 0.683 | 0.717 |

**Notes：**Logistic regression, LR;

***P* < 0.001.
